# Identification of a CARM1 Inhibitor with Potent *In Vitro* and *In Vivo* Activity in Preclinical Models of Multiple Myeloma

**DOI:** 10.1038/s41598-017-18446-z

**Published:** 2017-12-21

**Authors:** Allison E. Drew, Oscar Moradei, Suzanne L. Jacques, Nathalie Rioux, Ann P. Boriack-Sjodin, Christina Allain, Margaret Porter Scott, Lei Jin, Alejandra Raimondi, Jessica L. Handler, Heidi M. Ott, Ryan G. Kruger, Michael T. McCabe, Christopher Sneeringer, Thomas Riera, Gideon Shapiro, Nigel J. Waters, Lorna H. Mitchell, Kenneth W. Duncan, Mikel P. Moyer, Robert A. Copeland, Jesse Smith, Richard Chesworth, Scott A. Ribich

**Affiliations:** 1 Epizyme, Inc., Cambridge, Massachusetts, USA; 2 Epigenetics Discovery Performance Unit, Oncology R&D, GlaxoSmithKline, Collegeville, Pennsylvania, USA

## Abstract

CARM1 is an arginine methyltransferase with diverse histone and non-histone substrates implicated in the regulation of cellular processes including transcriptional co-activation and RNA processing. CARM1 overexpression has been reported in multiple cancer types and has been shown to modulate oncogenic pathways in *in vitro* studies. Detailed understanding of the mechanism of action of CARM1 in oncogenesis has been limited by a lack of selective tool compounds, particularly for *in vivo* studies. We describe the identification and characterization of, to our knowledge, the first potent and selective inhibitor of CARM1 that exhibits anti-proliferative effects both *in vitro* and *in vivo* and, to our knowledge, the first demonstration of a role for CARM1 in multiple myeloma (MM). EZM2302 (GSK3359088) is an inhibitor of CARM1 enzymatic activity in biochemical assays (IC_50_ = 6 nM) with broad selectivity against other histone methyltransferases. Treatment of MM cell lines with EZM2302 leads to inhibition of PABP1 and SMB methylation and cell stasis with IC_50_ values in the nanomolar range. Oral dosing of EZM2302 demonstrates dose-dependent *in vivo* CARM1 inhibition and anti-tumor activity in an MM xenograft model. EZM2302 is a validated chemical probe suitable for further understanding the biological role CARM1 plays in cancer and other diseases.

## Introduction

Reversible methylation of histones and other proteins is a key post-translational modification process involved in cellular development and tumorigenesis. CARM1 (coactivator-associated arginine methyltransferase 1, also known as PRMT4), catalyzes the transfer of up to two methyl groups to arginine residues on protein substrates^1^. Eleven mammalian protein arginine methyltransferases (PRMTs) have been identified to date and are classified by their mechanism of action into two types^2^. Each type is defined by its ability to transfer one or two methyl groups to the nitrogen atoms of the guanidinium side chains of arginine residues using S-adenosylmethionine (SAM) as the methyl donor. Addition of methyl groups by Type I PRMTs, (CARM1, PRMT1, PRMT3, PRMT6 and PRMT8) can result in both ω-N^G^-monomethyl (Rme1) and asymmetrical ω-N^G^,N^G^-dimethylarginine (aDMA), while type II PRMTs (PRMT5, PRMT7, and PRMT9), catalyze the formation of ω-N^G^-monomethyl (Rme1) and/or symmetrical ω-N^G^,N’^G^-dimethylarginine (sDMA). PRMT2, PRMT10 and PRMT11 do not possess known catalytic arginine methylation activity^2^.

CARM1 has been reported to methylate over 300 histone and non-histone substrates through which it can mediate effects on many cellular processes including transcriptional co-activation, RNA splicing and processing, control of cell cycle, and cellular differentiation^3^. The substrate motifs preferred by CARM1 are distinct from those preferred by other type I RMTs including PRMT1^1^. In contrast to the GGRGG methylation motif preferred by PRMT1, CARM1 substrates do not contain the RGG motif but do contain proline-rich sequences^3^. CARM1’s histone substrates are also distinct from those of other RMTs and have been reported to include H3R17 and H3R26^4^. H3R17 methylation is primarily thought to promote active transcription through the recruitment of transcriptional elongation complexes and other mechanisms^5^. Consistent with its role as a transcriptional co-activator, its non-histone substrates include nuclear receptors and nuclear receptor-associated co-activators such as SRC-3^6^, NCOA2^7^, and EP300^8^, as well as members of the SWI/SNF chromatin remodeling complex^9^. CARM1 also plays a multi-faceted role in the regulation of post-transcriptional processing and turnover through the methylation of proteins such as PABP1 and SmB^1,10,11^. CARM1 may therefore impact gene expression at multiple levels, both as a direct regulator of transcription as well as through modification of post-transcriptional RNA processing.

CARM1 overexpression has been reported in many cancer types including breast^12^, prostate^13^, and liver^14^ and a role for CARM1 in oncogenesis in these and other cancer types has been proposed, though its mechanism is not clear^9,15,16^. Originally identified as a co-activator of steroid hormone receptor-mediated transcription, CARM1 has been shown to interact with several nuclear receptors including the estrogen and androgen receptors, and it may mediate oncogenic effects in cancers driven by these pathways^17^. CARM1 has been reported to methylate the transcription factor RUNX1, resulting in a methyl-RUNX1-dependent repressor complex that blocks myeloid differentiation in AML^18^. It has also been suggested that CARM1 may impact the balance between oxidative phosphorylation and aerobic glycolysis in cancer cells through methylation of substrates such as PKM2^19^.

Although an attractive potential target for anti-cancer therapy, there are no reports of suitable CARM1 inhibitors to test the role of its catalytic activity in both *in vitro* as well as in *in vivo* tumor xenograft models. To date, there have been several publications describing small molecule chemical modulators of CARM1^20–27^. Although some of these compounds report inhibition of the CARM1 enzyme with IC_50_ values in the double-digit nanomolar range, no CARM1-selective compounds exhibited effects in non-engineered cell lines, or at concentrations less than 5 µM. A dual CARM1/PRMT6 inhibitor has been shown to inhibit the methylation of the reported CARM1 substrate MED12 with a cellular IC50 value of 1.4 µM^28^. Recently, two selective cell active CARM1 inhibitors have been reported (http://www.thesgc.org/chemical-probes/SKI-73, http://www.thesgc.org/chemical-probes/TP-064), but we are aware of no publications detailing their activity. To our knowledge, none of these compounds has demonstrated target engagement or functional effects *in vivo*. Cell-active CARM1 inhibitors are needed to better understand the implications of pharmacological inhibition of CARM1 and to discover and develop novel agents with potential for anti-cancer therapy.

In this report, the discovery of a potent and selective CARM1 inhibitor EZM2302 is described. EZM2302 demonstrates *in vitro* anti-proliferative activity consistent with specific methyl mark inhibition; displays dose-dependent inhibition of CARM1 substrate methylation *in vivo*; and induces growth inhibition of human multiple myeloma tumor xenografts in mice after oral dosing. EZM2302 is therefore an appropriate tool compound to further test the role of CARM1 across other oncology indications in which it has been implicated.

## Results

### Discovery of selective CARM1 inhibitor EZM2302

**Figure 1:**
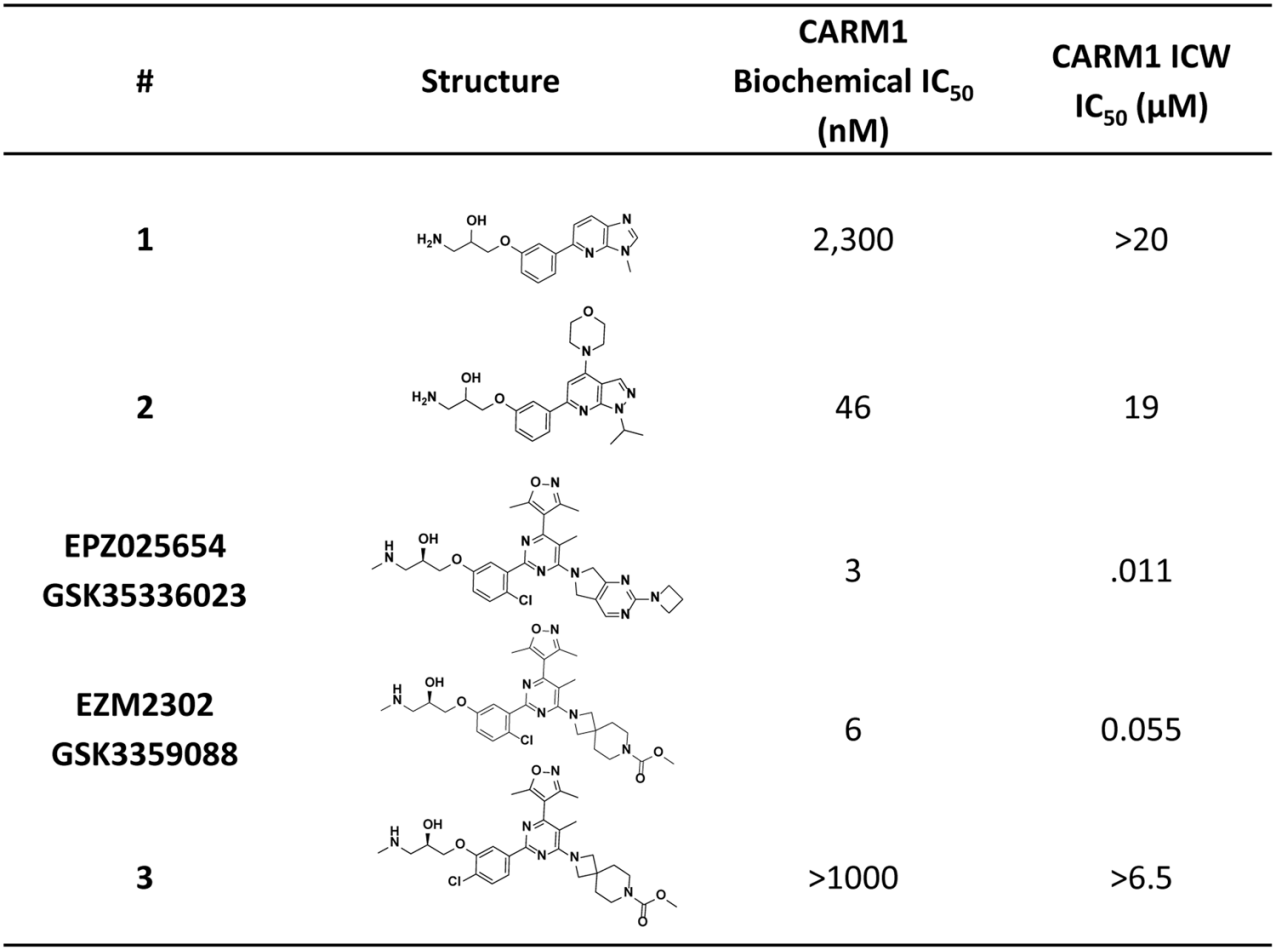
Compound structures and biochemical and cell data for CARM1 inhibitors.

**Figure 2:**
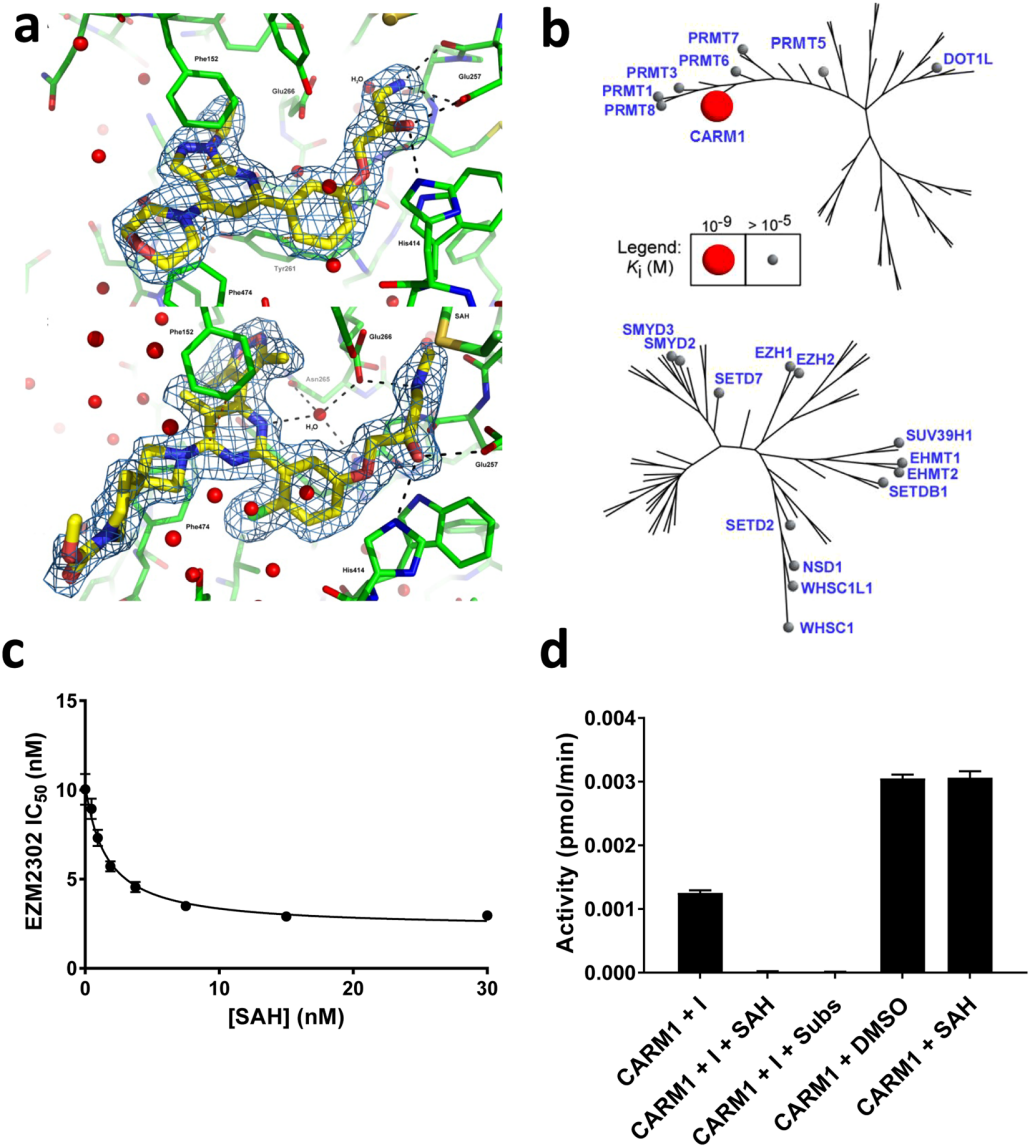
EZM2302 binds to CARM1 and is a selective inhibitor of CARM1 activity. (**a**) Structure of **2** (top) or EZM2302 (bottom) (yellow) in complex with CARM1 (green). Electron density (2Fo-Fc, 1σ for the compound is shown. Hydrogen bonds are indicated as black dashes; Π interactions are indicated with orange dashes; water molecules are depicted as spheres. (**b**) Ligand affinity maps of EZM2302 across the family trees of human arginine methyltransferases and lysine methyltransferase enzymes show EZM2302 is a selective and potent inhibitor of CARM1. (**c**) Synergy of CARM1 inhibition by EZM2302 with SAH. IC_50_ values for EZM2302 were determined at increasing concentrations of SAH. Data were fit using the noncompetitive Cheng-Prusoff and the Yonetani-Theorell equations as shown in the Supplementary Methods. Potency of inhibition by EZM2302 increases with SAH concentrations. (**d**) EZM2302 inhibition of CARM1. CARM1 was preincubated with excess EZM2302 (I) in the presence and absence of SAH and substrates (Subs). The CARM1 complexes were purified by gel filtration using 0.5 mL 7 K MWCO Zeba columns (Peirce) and CARM1 activity was tested.

Compound **1** (Fig. 1) was identified as a CARM1 inhibitor through rationale design based on inhibitors of other arginine methyltransferases^29–49^. It exhibited an IC_50_ value of 2.3 ± 0.8 μM (n = 3) and further ligand design was based on its structure. Compound **2** was 50-fold more potent against CARM1 than compound **1**, with an IC_50_ of 46 ± 9 nM (n = 2) and more than 100-fold selective for CARM1 over 20 other diverse HMTs in the Epizyme enzyme panel. X-ray crystallography of compound **2** revealed the compound was bound in the peptide substrate binding site (Fig. 2a, top). The 1-amino-3-phenoxypropan-2-ol moiety engaged in direct or water mediated hydrogen bonds with Glu257, His414, and Glu266. The benzene ring formed an edge-face interaction with Tyr261 and the pyrazolopyridine ring system formed π-stacking interactions with both Phe152 and Phe474. The isopropyl and morpholino groups also made van der Waals interactions with CARM1 residues. Interestingly, the structure unambiguously showed SAH bound in the nucleotide binding site despite the addition of SAM during crystallization. The structure confirmed the importance of the 1-amino-3-phenoxypropan-2-ol group and revealed areas of the molecule that could be further optimized.

Significant biochemical and cellular potency improvements were achieved after several rounds of SAR optimization, and thus compound EPZ0025654 (also known as GSK3536023) was identified as a suitable tool molecule for *in vitro* studies; however, without satisfactory pharmacokinetic profiles in mice and rats. Several rounds of structural refinements that focused on improving ADME parameters led to the synthesis of EZM2302 which was found to be a potent inhibitor in the biochemical assay (IC_50_ = 6 ± 3 nM, n = 21) and maintained its selectivity profile over other histone methyltransferases (Fig. 2b). EZM2302′s favorable physiochemical properties and ADME profile suggested its potential as an *in vitro* and *in vivo* tool compound. Finally, EPZ029751 was generated with a change of the substitution pattern of the benzene-chloro group. This compound has similar physiochemical properties to EZM2302 but much weaker biochemical potency against CARM1 and was used as a cell inactive control compound.

### Characterization of the Binding Mode of EZM2302

The crystal structure of EZM2302 was solved in CARM1 in complex with SAH and revealed subtle but important changes in the binding mode of the ligand and the protein when compared to earlier compounds. Overall, the position of EZM2302 is shifted 0.4–0.8 Angstroms when compared to compound **2**, resulting in an extensive, water mediated hydrogen bond network between the pyrimidine core and the protein (Fig. 2a, bottom). The addition of the methyl group to the 1-amino-3-phenoxypropan-2-ol moiety alters the vector of the methyl amine which results in a direct hydrogen bond to Glu266 and displacement of a water molecule. The chloro substitution of the benzene ring is accommodated with only minor changes to the protein and π-stacking interactions with Tyr261 and Phe152, and Phe474 are maintained. The dimethylisoxazole moiety makes van der Waals contacts with of the slide chains of Glu192, Tyr261 and Lys470. The spirocyclic moiety makes additional van der Waals interactions with Phe474 while the majority of the substituent is in a solvent exposed region.

### Inhibition of CARM1 by EZM2302 is synergistic with SAH

To better understand the mechanism of inhibition of CARM1 by EZM2302, substrate competition studies were performed. Inhibition of CARM1 by EZM2302 was evaluated at increasing concentrations of SAM or peptide substrate. When SAM was varied from 0.2*K*_M_ to 10*K*_M_, EZM2302 IC_50_ values varied less than 3-fold (Supplementary Fig. S1). This noncompetitive pattern of inhibition with respect to SAM is consistent with EZM2302 binding the arginine pocket of CARM1. Varying peptide concentrations also produced a noncompetitive pattern with *K*_i_ = 25 ± 3 nM and α = 0.2 (Supplementary Fig. S1). Based on the crystal structure, EZM2302 was expected to be competitive with peptide, however, the difference may be explained by peptide binding through exosite interactions in the presence of inhibitor^50,51^ or by other factors such as time-dependent inhibition^52,53^.

Accordingly, time dependence of CARM1 inhibition was investigated. When potency was monitored at increasing reaction times, the IC_50_ values decreased exponentially from 129 nM and reached a plateau at 11 nM (Supplementary Fig. S1). These results suggest that the inhibitor is binding to an enzyme-substrate complex or an enzyme conformation formed over the course of substrate turnover. IC_50_ values for compounds in this series were measured routinely at 120 minutes to ensure potency reached a plateau.

To further investigate the binding mode of EZM2302, inhibition of CARM1 was examined in a dual inhibitor study. CARM1 inhibition was measured at varying concentrations of both EZM2302 and SAH. Since SAH binds the SAM pocket, EZM2302 should not be mutually exclusive or competitive with SAH. Figure 2c shows that EZM2302 IC_50_ values decrease hyperbolically with increasing SAH and were fit with an α value of 0.2 demonstrating synergy of inhibition between EZM2302 and SAH. Synergy indicates that these compounds bind different pockets in CARM1, in agreement with the crystal structure. In fact, EZM2302 is only found in CARM1 crystals with SAH.

### EZM2302 forms a long-lived inhibitory complex with CARM1

Reversibility of CARM1 inhibition by EZM2302 was examined by gel filtration of the enzyme-inhibitor complex. Briefly, CARM1 was incubated with a large excess of EZM2302 in the presence or absence of SAH or substrates. Small molecules were subsequently removed by gel filtration and CARM1 activity was measured. Reversible inhibitors will recover full activity as demonstrated for SAH alone in Fig. 2d, whereas irreversible inhibitors will remain inactive. CARM1 activity could be partially rescued by gel filtration after incubation with EZM2302 alone though no rescue was observed after CARM1 and EZM2302 were incubated with either SAH or substrates (SAM + peptide). The addition of SAH or substrates appears to increase the extent of inhibition by EZM2302. The EZM2302/SAH/CARM1 complex was tested over 7 days and no activity was detected, indicating that EZM2302 forms a long-lived complex with CARM1 in the presence of SAH, which is consistent with results from the crystal structure.

### EZM2302 Inhibits Cellular PABP1 and SMB Methylation *in vitro*

**Figure 3:**
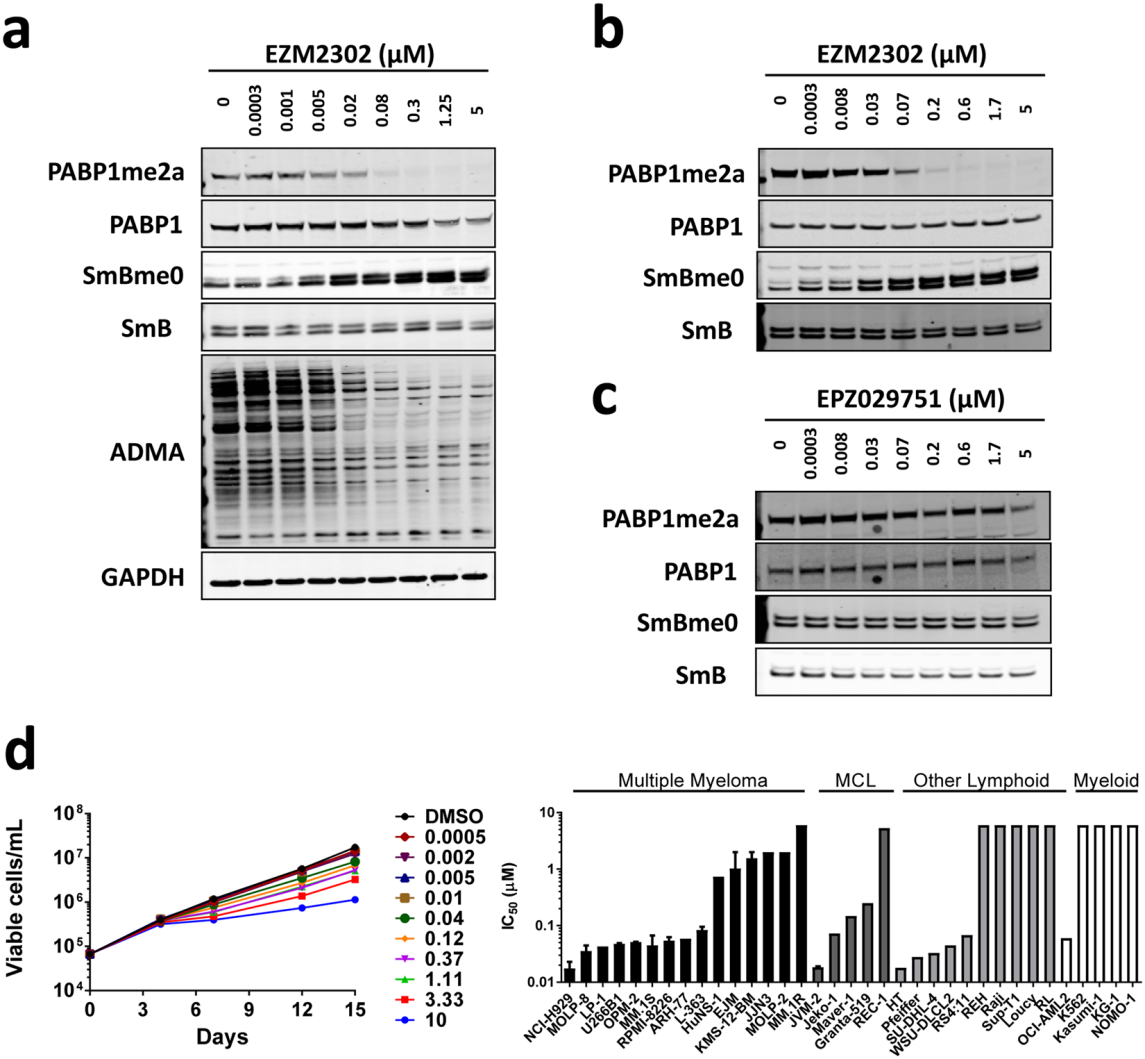
Effects of EZM2302 on cellular target inhibition and proliferation. (**a**) Concentration-dependent inhibition of cellular asymmetric dimethyl arginine substrates after four days of EZM2302 treatment in RPMI-8226 cells. Cells were treated with a dose-titration of 0.0003 to 5 µM compound. (**b**) Concentration-dependent inhibition of cellular asymmetric dimethyl arginine substrates after four days of EZM2302 treatment in NCI-H929 cells. NCI-H929 IC_50_ values were calculated as 0.009 µM (PABP1me2a), 0.031 µM (SmBme0). (**c**) Minimal effects of compound **3** on cellular target inhibition. Cellular asymmetric dimethyl arginine substrates were minimally impacted after four days of compound **3** treatment in RPMI-8226 cells. Cells were treated with a dose-titration of 0.0003 to 5 µM compound. IC_50_ values were greater than 5 µM for PABP1me2a and SmBme0. (**d**) Inhibition of proliferation by EZM2302 *in vitro* over 14 days in culture. Cells were counted and replated at the original seeding densities on days 4, 7, and 11. Each point represents the mean for each concentration (n = 3). Relative and absolute IC_50_ values were calculated in GraphPad Prism (non-linear regression analysis, top of the curves fixed to 100%) for each line using split-adjusted cell counts at day 14. Left panel: Growth curves for RPMI-8226 cell line, each point represents the mean of three replicates. Right panel: Day 14 IC_50_ values for 36 hematopoietic cell lines. Images in (**a**,**b** and **c**) have been cropped. Uncropped images are presented in Supplementary Fig. S2.

To assess the cellular activity of EZM2302 in *vitro*, we quantified changes in cellular methylation levels upon treatment with CARM1 inhibitor. The effect of EZM2302 treatment on cellular methylation was tested by immunoblot in the multiple myeloma (MM) cell line RPMI-8226 (Fig. 3a, Supplementary Fig. S2). Methylation changes were measured at the well-characterized CARM1 substrates PABP1 and SmB^1,10^. Changes in overall levels of asymmetric dimethyl arginine (aDMA) were assessed with an antibody that detects a generic aDMA motif. Levels of methylated analytes were quantified by densitometry and ratios of methylated to total protein were calculated to determine the cell biochemical IC_50_ values. A monoclonal SmB antibody was validated that selectively detects the unmethylated form of the protein (SmBme0). This antibody detects unmethylated SmB peptides but not methylated SmB peptides by western blot, and detects a robust increase in unmethylated SmB after shRNA CARM1 knockdown or treatment with a CARM1 inhibitor (Supplementary Fig. S3,S4). Ninety-six-hour EZM2302 treatment led to a concentration-dependent decrease in methylation of PABP1 (IC_50_ = 0.038 ± 0.015 µM, N = 3) and SmB (increased levels of SmBme0, EC_50_ = 0.018 ± 0.007 µM, N = 3), as well as in multiple aDMA bands (IC_50_ = 0.009 µM). Similar results were also observed in the NCI-H929 (Fig. 3b and Supplementary Fig. S2) and U266B1 MM cell lines (Supplementary Fig. S5,S6). No robust changes in methylation were observed after treatment of RPMI-8226 with the inactive analog, EPZ029751 (Fig. 3c and Supplementary Fig. S2). The effects of CARM1 inhibition on cellular histone methylation at the putative CARM1 substrates H3R17 and H3R26 were also evaluated by performing western blot analysis on whole cell lysates. Ninety-six-hour EZM2302 treatment in RPMI-8226 cells did not result in any significant decreases in global histone methylation levels as detected by western blot (Supplementary Figs S7 and S8).

### EZM2302 inhibits the *in vitro* proliferation of multiple hematopoietic cell lines

The anti-proliferative effect of EZM2302 treatment was assessed in a panel of solid tumor cell lines representing indications in which CARM1 has been hypothesized in the literature to play an oncogenic role, including breast, colorectal, and prostate carcinoma. As the anti-proliferative effects of the inhibitors of epigenetic modifiers are known to manifest over several days, we assessed functional effects of EZM2302 using a long term proliferation (LTP) assay that allowed the measurement of cell growth over 14 (suspension) or 15 (adherent) days^54,55^. Minimal activity (IC_50_ > 10 µM) was observed in 12/12 tested cell lines (Supplementary Table S2) suggesting that CARM1 methyltransferase activity does not drive proliferation in these lines. However, when a diverse set of 36 hematopoietic cancer cell lines was tested a range of anti-proliferative effects was observed with absolute IC_50_ values ranging from 0.015 to >10 µM (Fig. 3d). There was no relationship between the level of CARM1 transcript expression and the sensitivity to EZM2302 treatment or between the level of CARM1 protein and EZM2302 when examined in a subset of MM cell lines (Supplementary Fig. S9). Potent anti-proliferative effects were most frequent in the tested multiple myeloma cell lines, with day 14 IC_50_ values of less than 100 nM in 9 of 15 cell lines. EZM2302 appears to act in a cytostatic, not cytotoxic, fashion in these cell lines as can be seen by the growth curves in the RPMI-8226 cell line which are representative of those observed in other sensitive multiple myeloma cell lines (Fig. 3d). Similar results were observed in a 6-day proliferation assay, although IC_50_ values were much higher than was observed in the 14-day assay (Supplementary Table S3). This is consistent with the delayed kinetics of an histone methyltransferase inhibitor, which require multiple days to reduce cellular methyl mark, elicit transcriptional effects, and inhibit cellular proliferation^54,56,57^.

### EZM2302 exhibits appropriate pharmacokinetic properties for *in vivo* studies

**Figure 4:**
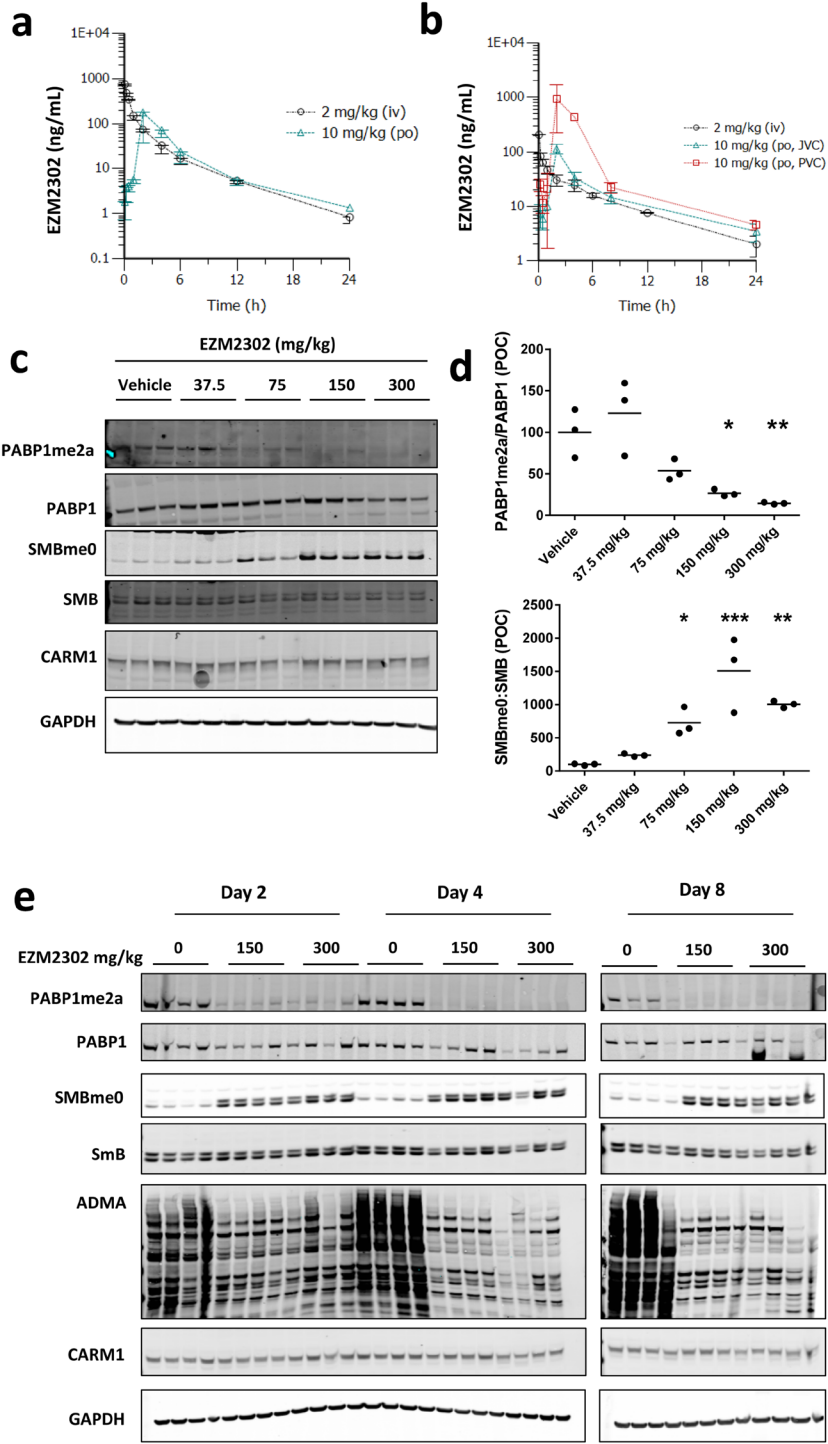
EZM2302 is orally available with potent *in vivo* CARM1 inhibition. (**a**) Preclinical PK of EZM2302 in (**a**), CD-1 mouse and (**b**) Sprague-Dawley rat with or without jugular- and portal-vein cannulation (JVC and PVC). Data are shown graphically as plasma concentration vs. time profile of mean ± SD (n = 3) following i.v. bolus or oral gavage administration. (**c**) Methyl mark changes induced by twice daily (BID) administration of EZM2302 for 7 days at 150 and 300 mg/kg in untumored CB-17 SCID mice. Compound administration was stopped on day 7, and liver tissue was harvested for PD analysis. (**d**) Each point represents the ratio of PABP1me2a to PAPB1 or SmBme0 to SmB normalized to the vehicle control, measured by western blot. The horizontal lines represent group mean values. Dose groups that showed a statistically significant inhibition are indicated (*p < 0.05, **p < 0.01, ***p < 0.001, ****p < 0.0001, versus vehicle, 1-way ANOVA with a Dunnett’s post-test) P values compared to vehicle were: PABP1me2a: 37.5 mg/kg p = 0.634, 75 mg/kg p = 0.137, 150 mg/kg p = 0.016, 300 mg/kg p = 0.006, SMBme0: 37.5 mg/kg p = 0.092, 75 mg/kg p = 0.052, 150 mg/kg p = 0.0003, 300 mg/kg p = 0.007. (**e**) CARM1 target inhibition in RPMI-8226 xenograft tumor tissue collected from mice euthanized after 2, 4, and 8 days of BID dosing at 150 and 300 mg/kg EZM2302. Images in C and E have been cropped. Uncropped images are presented in Supplementary Figs S9 and S10.

EZM2302 was stable in human hepatocytes (CL <3 mL/min/kg), and moderately bound to human, mouse and rat plasma proteins with a mean fraction unbound of 0.66, 0.46 and 0.74, respectively. In mouse and rat, the plasma clearance (CL) was 43 and 91 mL/min/kg, respectively (Table 1, Fig. 4a,b). In rat, a low level of binding to erythrocytes was observed, whereas EZM2302 did not show blood partitioning in mouse, therefore, blood CL was equivalent in both species. Although rats showed a moderate mean bioavailability (F), the fraction of the dose absorbed from the gastro-intestinal tract (Fa*Fg) was much higher at 81% as measured by performing JVC-PVC rat PK experiment, reflective of EZM2302 high permeability. Therefore, EZM2302 is orally bioavailable and amenable to *in vivo* studies.

**Table 1:** The preclinical pharmacokinetics of EZM2302 determined in mouse and rat.

**Parameter**	**CD-1 mouse**		**Sprague-Dawley rat**	
	**IV Bolus**	**PO**	**IV Bolus**	**PO**
Dose (mg/kg)	2	10	2	10
Blood:plasma ratio	1.2	1.2	2.6	ND
C_max_ (ng/mL)		177		113 ± 22.4
T_max_ (h)		2.00		2.00
AUC_0-last_ (ng.h/mL)	767	568	352 ± 30.6	453 ± 89.3
AUC_0-inf_ (ng.h/mL)	772	577	372 ± 43.3	487 ± 102
t_1/2_ (h)	4.22	4.55	6.21 ± 1.65	6.64 ± 1.41
V_ss_ (L/kg)	6.53		35.6 ± 1.30	
CL (mL/min/kg)	43.2		90.5 ± 10.5	
F_a_*F_g_ (%)		ND		80.7
F (%)		15.0		26.2 ± 5.45

Values reported as mean, n = 9/group, n = 3/timepoint for mouse and mean ± SD, n = 3/group, for rat. PK data analysis was performed using noncompartmental analysis and WinNonlin Phoenix 6.2 (or higher). ND: Not determined.

EZM2302 did not inhibit CYP1A2, CYP2C9, CYP2C19, CYP2D6 or CYP3A4 when tested up to 25 µM and showed an IC_50_ > 100 µM against hERG channel current inhibition. In addition, specificity was also demonstrated in an ExpresSProfile™ (CEREP, Redmond, WA) screen of 55 receptors, ion channels and transporters. In this screen, EZM2302 applied at 10 μM failed to show >40% inhibition or stimulation in any of the assays, with the exception of the rat recombinant kappa-opioid (KOP) receptor binding assay which displayed 69% inhibition. The IC_50_ was determined to be 3.3 μM for this receptor. Furthermore, 10 μM EZM2302 did not show inhibition >30% against 40 protein kinases, lipid kinases and kinase mutants in the functional KinaseProfiler™ screen (Millipore, Dundee, UK). These data suggest that EZM2302 is generally inactive against a broad array of *in vitro* targets, thus decreasing the risk of off-target effects *in vivo*.

### Anti-Tumor Effects of EZM2302 in the Treatment of Multiple Myeloma Xenografts

A dose range-finding (DRF) study conducted for 7 days with twice daily (BID) oral dosing of EZM2302 in CB-17 SCID mice at 37.5–300 mg/kg showed that all doses were well-tolerated with minimal body weight loss (Supplementary Fig. S10). Peripheral tissue was collected at day 7 and levels of PABP1 and SmB methylation were assessed. Methylation was robustly inhibited in a dose-dependent manner in liver (Fig. 4c,d and Supplementary Fig. S11) and lung (data not shown) tissue, indicating that EZM2302 is suitable for *in vivo* studies of CARM1 inhibitors. Studies were initiated in a RPMI-8226 xenograft model to determine the relationship between methyl mark pharmacodynamics (PD) and tumor growth inhibition (TGI). To understand the kinetics of *in vivo* target inhibition, levels of PABP1, SmB, and aDMA methylation were assessed in tumor tissue after 2, 4, and 8 days of EZM2302 treatment at 150 and 300 mg/kg BID, based on the tolerability of these doses in the DRF. Inhibition of PAPB1me2a and aDMA and induction of SmBme0 were robustly observed within two days of dosing, with maximal changes in methylation observed at day 4 in both dose groups (Fig. 4e and Supplementary Fig. S11).

**Figure 5:**
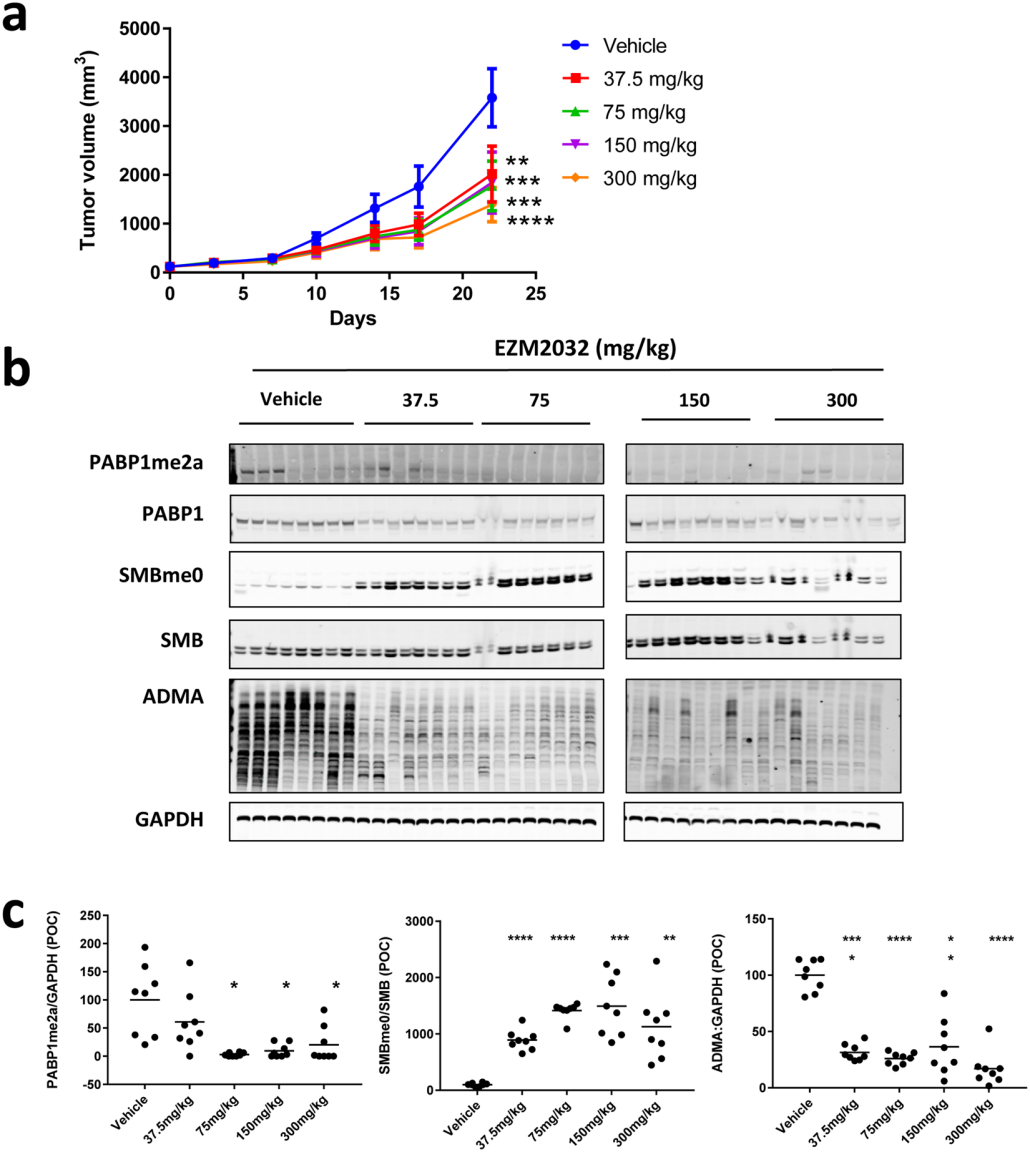
EZM2302 shows dose dependent target engagement and tumor growth inhibition *in vivo*. (**a**) Anti-tumor activity in RPMI-8226 xenograft model induced by twice daily (BID) administration of EZM2302 for 21 days at the indicated doses (N = 8, Mean value ± SEM). Tumor growth rates were significantly reduced at all dose groups (*p < 0.05, **p < 0.01, ***p < 0.001, ****p < 0.0001, versus vehicle, 2-way ANOVA with a Dunnett’s post-test). P values compared to vehicle were: 37.5 mg/kg p = 0.004, 75 mg/kg p = 0.0008, 150 mg/kg p = 0.007, 300 mg/kg p = 0.0001. Compound administration was stopped on day 21, and tumors were harvested for PD analysis. (**b**) CARM1 target inhibition in RPMI-8226 xenograft tumor tissue collected from mice euthanized after 21 days of BID dosing. (**c**) Each point represents the ratio of PABP1me2a to GAPDH, aDMA to GAPDH, or SmBme0 to SmB normalized to the vehicle control, measured by WB. Dose groups that showed a statistically significant inhibition are indicated (*p < 0.05, **p < 0.01, ***p < 0.001, ****p < 0.0001, versus vehicle, 1-way ANOVA with a Dunnett’s post-test). P values compared to vehicle were: PABP1me2a: 37.5 mg/kg p = 0.603, 75 mg/kg p = 0.011, 150 mg/kg p = 0.020, 300 mg/kg p = 0.036, SMBme0: 37.5 mg/kg p = 0.0001, 75 mg/kg p = 0.0001, 150 mg/kg p = 0.0004, 300 mg/kg p = 0.005, ADMA: 37.5 mg/kg p = 0.0001, 75 mg/kg p = 0.0001, 150 mg/kg p = 0.0021, 300 mg/kg p = 0.0001. Images in (**b**) have been cropped. Uncropped images are presented in Supplementary Fig. S11.

Tumor growth inhibition studies were performed in SCID mice bearing subcutaneous RPMI-8226 xenografts with twice daily (BID) oral dosing on four dose groups: 37.5, 75, 150 and 300 mg per kilogram (mg/kg). After 21 days of continuous dosing, animals were euthanized to collect blood and tissues for pharmacokinetic and methyl mark analysis. EZM2302 showed dose-dependent exposure and tumor growth inhibition (TGI) after 21 days in the RPMI-8226 xenograft model (Fig. 5a). Tumors in all EZM2302 dose groups measured on day 21 showed significant decreases in tumor growth compared to vehicle (2-way ANOVA compared to Vehicle, Dunnett’s post-test). Tumor growth inhibition ranged from 45% in the 37.5 mg/kg dose group to 63% in the 300 mg/kg dose group. Though some body weight loss was noted in the 300 mg/kg group, EZM2302 was well tolerated at 150 mg/kg and below with minimal bodyweight loss and no other clinical observations (Supplementary Fig. S5). RPMI-8226 xenograft tumors collected on day 21 showed a dose-dependent decrease in methylation at all tested CARM1 substrates (Fig. 5b,c and Supplementary Fig. S12). A statistically significant increase in unmethylated form of SmB (SmBme0) was detected at all dose groups, from an 8-fold increase at 37.5 mg/kg to a 14-fold increase at 150 mg/kg. aDMA levels were likewise significantly decreased at all dose groups, a maximal inhibition of 65% was observed at the 75 mg/kg dose group. Levels of both total and methylated PABP1 were difficult to detect in xenograft tissues. Methylated PABP1 was undetectable in most animals in the 75 mg/kg and higher dose groups. Similar inhibition of methylation of CARM1 substrates was observed after 21-day treatment of a second multiple myeloma xenograft mouse model, NCI-H929, but in this case no significant anti-tumor effects were observed (data not shown).

## Discussion

A growing body of literature supports the important role of lysine and arginine methylation of protein substrates in both normal and disease biology. Studies elucidating the contribution of aberrant lysine methylation to oncogenesis have been greatly facilitated by the availability of molecular probes for this target class, and first-in-class inhibitors of lysine methyltransferases are currently being tested in the clinic. An understanding of the potential utility of arginine methyltransferase inhibitors is less developed, in part due to the lack of available tool compounds. The first small molecule inhibitor of an arginine methyltransferase with *in vivo* target engagement and efficacy has only recently been demonstrated^58^. Here, we describe the cell-potent, orally bioavailable, and selective CARM1 inhibitor, EZM2302. To our knowledge, this is the first described CARM1 inhibitor that has demonstrated both *in vivo* target engagement and efficacy in xenograft tumor models. This study is also the first to our knowledge in which a potential role for CARM1 as a driver in multiple myeloma has been reported.

EZM2302 demonstrates robust *in vitro* target engagement at the previously-reported and well-validated CARM1 substrates PABP1 and SmB. However, inhibition of CARM1 by EZM2302 also caused a large reduction in the signal from an aDMA antibody, suggesting that there are many additional CARM1 non-histone substrates. This would be consistent with the recent proteomic analysis of CARM1, which identified >300 unique aDMA sites on 138 different proteins^3^. The availability of a potent and selective CARM1 inhibitor presents opportunities for further exploration of these novel CARM1 substrates in both *in vitro* and *in vivo* studies. Interestingly, in cells EZM2302 does not show a global reduction in methylation at the reported CARM1 histone methyl marks, H3R26 or H3R17 (Supplementary Figs S7 and S8), nor do we see a reduction at these marks following shRNA knockdown (data not shown). Global changes at this mark have been reported only in embryonic fibroblasts from CARM1-knockout mice^10^. Modulation following CARM1 inhibition or knockdown may occur in a more site-specific manner and may not be detectable in a whole cell lysate.

While other small molecule CARM1 inhibitors have been described, including compounds that have demonstrated CARM1 binding in a crystal structure^26^ potency and pharmacokinetic properties have not been shown to be appropriate for *in vitro* and *in vivo* target validation studies. Demonstration of inhibition of CARM1-methylated substrates with these compounds has been achieved only at doses of greater than 5 µM, where selectivity against other enzymes may be a concern, or in engineered cell systems. Therefore, previously, limited data were available to suggest that CARM1 inhibition resulted in a dose-dependent effect on cellular proliferation. The majority of these studies were conducted in epithelial-derived cancer cell lines, based on reports of CARM1 overexpression in these indications. Though CARM1 overexpression has been reported in multiple cancer types, its role in oncogenesis is not fully understood. In our hands, no relationship was observed between levels of CARM1 expression and sensitivity to CARM1 inhibition with EZM2302. *In vitro* experiments using genetic knockdown of CARM1 have shown anti-proliferative effect of knockdown in several cancer cell lines. These experiments have suggested a potential role for CARM1 in modulating WNT-induced expression of β-catenin genes in colorectal cancer^59^, modulating transcriptional activation of the androgen receptor in prostate cancer^60^, and NCOA2-dependent regulation of estrogen-mediated transcriptional activation in breast cancer^7^. Treatment of colorectal, prostate and breast cancer cell lines with the CARM1 inhibitor EZM2302 had no anti-proliferative effect (Supplementary Table 2), suggesting that CARM1 catalytic activity is not necessary for proliferation in these indications, though it does not rule out a potential non-catalytic role of CARM1, such as a protein scaffolding function. Indeed, expression of a catalytic-inactive CARM1 mutant reduces but does not eliminate transcriptional coactivation, suggesting that non-catalytic mechanisms are also at play^61,62^. Further studies with inhibitors of CARM1 methyltransferase activity such as EZM2302 will enable a more detailed study of the importance of these different mechanisms of action to oncogenesis.

This work demonstrates anti-proliferative effects of CARM1 inhibition by EZM2302 treatment in hematopoietic cancer cell lines, which were most frequently observed in multiple myeloma cell lines. We believe this is a novel finding, as no previous work to our knowledge suggests a role for CARM1 in multiple myeloma pathogenesis. Consistent with the *in vitro* proliferation data, we observed dose-dependent *in vivo* target engagement and tumor growth inhibition in the RPMI-8226 multiple myeloma xenograft model. A second multiple myeloma xenograft model (NCI-H929) did not respond to EZM2302 treatment. This heterogeneity in response was also observed in *in vitro* proliferation studies in a panel of multiple myeloma cell lines, and may be due in part to the heterogeneous genetic backgrounds of the different multiple myeloma cell line models. The mechanism of action that underlies the sensitivity to CARM1 inhibition will require further elucidation.

In summary, a novel series has been identified as CARM1 inhibitors. Multi-parametric chemical optimization resulted in compound EZM2302, which exhibited nanomolar biochemical activity against CARM1 that was well correlated with both cellular target engagement and *in vitro* anti-proliferative effect, and shows exquisite selectivity against other HMTs with no off-target activities. Treatment with EZM2302 resulted in anti-proliferative activity in multiple myeloma cell lines and dose-dependent tumor growth inhibition in a multiple myeloma subcutaneous tumor xenograft model in mice. Importantly, this is the first report to our knowledge that demonstrates a potential role for a CARM1 inhibitor in multiple myeloma. EZM2302 is a tool compound that can be used to further explore the biological role of CARM1 and understand the role of this enzyme in multiple myeloma and other oncology indications.

## Methods

### Compounds

The synthesis of all compounds have been reported elsewhere^63,64^; experimental procedures for the synthesis of EZM2302, EPZ025654, and EPZ029751 are in Supplementary Methods.

### Protein production

FLAG-CARM1 (amino acids 2–585)-His for biochemical studies was expressed in 293 F mammalian cells and purified using FLAG affinity chromatography. His-TEV-CARM1 (amino acids 134–479) for x-ray crystallography was produced using insect cells and purified using Ni affinity and size exclusion chromatography. Detailed methods can be found in Supplementary Methods.

### X-ray crystallography

Crystals of CARM1 (amino acids 134–479) were grown using vapor diffusion methods and compounds were soaked into pre-formed crystals. Crystallization and structure determination methods, including data collection and refinement statistics, can be found in Supplementary Methods and in Supplementary Table S1. Structures have been deposited in the Protein Data Bank with the following PDB codes: Compound **2**: 6ARV; EZM2302: 6ARJ.

### Histone methyltransferase assay

CARM1 activity was measured as previously reported for the histone methyltransferases PRMT1/6/8^65^ and PRMT5^58^. Briefly, CARM1 was preincubated with compounds for 30 minutes at room temperature before reactions were initiated. Final assay conditions were 0.25 nM CARM1, 30 nM ^3^H-S-adenosyl-methionine (SAM), and 250 nM biotinylated peptide in buffer containing 20 mM bicine, 1 mM tris(2-carboxyethyl)phosphine, 0.005% bovine skin gelatin and 0.002% Tween-20, pH 7.5. The assays were quenched by the addition of 300 μM unlabeled SAM. The quantity of ^3^H-labeled peptide produced was measured by Flashplate on a Topcount reader. Further details can be found in the Supplementary Methods.

### Cell line and antibodies

All internally tested cell lines were obtained from commercial sources and cultured in supplier’s recommended media. Cell lines were authenticated using the GenePrint 10 system STR multiplex assay (Promega) that amplifies 9 tetranucleotide repeat loci and Amelogenin gender determining marker. Antibodies: Cell Signaling Technology, CARM1 (C31G9, CS3379) PABP1 (CS4992), PABP1me2a (C60A10, CS3505), aDMA (D10A12E9, CS13076), Histone H3 (96C10, CS3638) EMD Millipore, GAPDH (6C5, MABE374), H3R26me1 (ABE14), Sigma-Aldrich, SmB (12F5, S0698), Abgent, SmB, (AP2959a), Abcam, PABP1 (10E10, ab6125), 21^st^ Century Biochemicals, H3R17me2a (custom formulation).

### *In vitro* compound treatment

Cultured cells in linear/log phase growth were split to a seeding density of 2e5 cells/mL in 2–20mLs of media, depending on the yield required at the end of the growth period. Compound was diluted in DMSO and added to each culture vessel with a final DMSO concentration of 0.2%. Cells were allowed to grow for 96 hours. At the conclusion of each treatment period, cells were harvested by centrifugation (5 minutes, 1200 rpm), and cell pellets were rinsed once with PBS before being frozen on dry ice pending further processing.

### Western blotting

Lysates were prepared from cell pellets, flash frozen tumor xenograft samples, or peripheral tissue in 1X RIPA buffer with 0.1% SDS. Quantification of Western blots was performed using infra-red emitting secondary antibodies and detection with an ODYSSEY infrared imaging system.

### *In vitro* Proliferation Assay

Long term (14–15 day) cell proliferation assays were performed using the method previously described with adjustments to initial seeding densities depending on growth characteristics for each cell line^54,55^. For 6 day proliferation studies, see Supplementary Methods.

### Pharmacokinetic Study in Mouse and Rat

All of the procedures related to animal handling, care and the treatment in this study were performed according to the guidelines approved by the Institutional Animal Care and Use Committee (IACUC) of Shanghai Chempartner following the guidance of the Association for Assessment and Accreditation of Laboratory Animal Care (AAALAC). Experimental protocols were approved by the IACUC of Shanghai Chempartner. Male CD-1 mice (n = 9/group, 3 per time-point) and male Sprague-Dawley rats (n = 3) were treated with a single dose of EZM2302 at 2 mg/kg by intravenous (i.v.) injection and 10 mg/kg by oral gavage administration (p.o.; mouse only), formulated in 5% dextrose in water, pH 3.5. An additional group of rats, cannulated in both the jugular and portal veins were dosed by oral gavage (10 mg/kg, in 0.5% methylcellulose in water). Approximately 110 μL of blood was taken from the animals by retro-orbital bleeding (mouse), tail vein (rat i.v.) or both jugular and portal vein sampling (rat p.o.) at pre-specified time intervals. The 2 h samples were split for parallel determination of blood and plasma concentration (*ex vivo* ratio). Blood samples were collected into K_2_-EDTA tubes and centrifuged to obtain plasma. Following protein precipitation, EZM2302 concentrations were determined by LC-MS/MS analysis and data were analyzed using Phoenix WinNonlin 6.2 or higher.

### ADME Assays

Hepatocyte stability, plasma protein binding and cytochrome P450 assays were performed using standard procedures. Details can be found in Supplementary Methods.

### *In vivo* xenograft studies

All of the procedures related to animal handling, care and the treatment in this study were performed according to the guidelines approved by the Institutional Animal Care and Use Committee (IACUC) of Shanghai Chempartner following the guidance of the Association for Assessment and Accreditation of Laboratory Animal Care (AAALAC). Experimental protocols were approved by the IACUC of Shanghai Chempartner. For the *in vivo* efficacy studies, there were 8 mice per dose group and each mouse was inoculated subcutaneously at the right flank. All cells were suspended in a 0.2 mL mixture of base media and Matrigel at 1:1 for tumor development. RPMI-8226 cells were inoculated at 5 × 10^6^ cells/mouse and treatment began when the mean tumor sizes reached 120 mm^3^ (28 days post-inoculation). CB-17 SCID Mice were assigned into groups using a randomized block design. EZM2302 or vehicle (0.5% methylcellulose in water) was administered orally BID at a dose volume of 37.5, 75, 150, or 300 mg/kg for 21 days. Body weights were measured twice a week for the duration of the study. Tumor size was measured twice weekly in two dimensions using a caliper, and the volume was expressed in cubic millimeters. Animals were euthanized 3 hours post-final dose, with blood and tissues collected for analysis.

### Data availability

The datasets generated during and/or analyzed during the current study are available from the corresponding author on reasonable request.

## Electronic supplementary material

Supplementary information
