# Supplementary material for: Identification of a CARM1 Inhibitor with Potent In Vitro and In Vivo Activity in Preclinical Models of Multiple Myeloma
Source: Sci Rep. 2017 Dec 21;7:17993. doi: 10.1038/s41598-017-18446-z (PMC5740082; doi:10.1038/s41598-017-18446-z)
Supplement: Supplementary file 1 — Supplementary information [file 41598_2017_18446_MOESM1_ESM.pdf]

Identification of a CARM1 Inhibitor with Potent *In Vitro* and *In Vivo* Activity in Preclinical Models of Multiple Myeloma.

Allison E. Drew[1*#], Oscar Moradei[1#], Suzanne L. Jacques[1], Nathalie Rioux[1], P. Ann Boriack-Sjodin[1], Christina Allain[1], Margaret Porter Scott[1], Lei Jin[1], Alejandra Raimondi[1], Jessica L. Handler[2], Heidi M. Ott[2], Ryan G. Kruger[2], Michael T. McCabe[2], Christopher Sneeringer[1], Thomas Riera[1], Gideon Shapiro[1], Nigel J. Waters[1], Lorna H. Mitchell[1], Kenneth W. Duncan[1], Mikel P Moyer[1], Robert A. Copeland[1], Jesse Smith[1], Richard Chesworth[1], Scott A. Ribich[1]

Affiliations: [1]Epizyme, Inc., Cambridge, Massachusetts, USA. [2]Cancer Epigenetics Discovery Performance Unit, Oncology R&D, GlaxoSmithKline, Collegeville, Pennsylvania.
[*]Corresponding author [#]Authors contributed equally.

SUPPLEMENTAL MATERIALS AND METHODS:

Compound synthesis:

Synthesis of (R)-1-(3-(4-(2-(azetidin-1-yl)-5,7-dihydro-6H-pyrrolo[3,4-d]pyrimidin-6-yl)-6-(3,5-dimethylisoxazol-4-yl)-5-methylpyrimidin-2-yl)-4-chlorophenoxy)-3-(methylamino)propan-2-ol (EPZ025654)

Step 1: Synthesis 2-(azetidin-1-yl)-6,7-dihydro-5H-pyrrolo[3,4-d]pyrimidine 2,2,2-trifluoroacetate: A solution of tert-butyl 2-(methylsulfonyl)-5,7-dihydro-6H-pyrrolo[3,4-d]pyrimidine-6-carboxylate (480 mg, 1.6 mmol) in DMF (3 mL), was treated with DIPEA (0.5 mL) followed by addition of azetidine hydrochloride salt (300 mg; 3.2 eq.). The mixture was placed in a capped vial and stirred at external temperature of 60 °C for 16 h, then diluted with ethyl acetate (80 mL), washed with water (50 mL x 3) and the organic phase concentrated under vacuum to render a light yellow solid. This crude material was dissolved in 4 mL of 90% aq. trifluoroacetic acid and the mixture was stirred at room temperature for 60 min. Removal of volatiles under vacuum rendered a yellow solid characterized as 2-(azetidin-1-yl)-6,7-dihydro-5H-pyrrolo[3,4-d]pyrimidine 2,2,2-trifluoroacetate was obtained (540 mg, ca 1.6 mmol, quant. yield). ESI-LCMS (m/z): 177.1 [M+H]+.

Step 2: Synthesis of tert-butyl (R)-(3-(3-(4-(2-(azetidin-1-yl)-5,7-dihydro-6H-pyrrolo[3,4-d]pyrimidin-6-yl)-6-(3,5-dimethylisoxazol-4-yl)-5-methylpyrimidin-2-yl)-4-chlorophenoxy)-2-((tert-butyldimethylsilyl)oxy)propyl)(methyl)carbamate: A reaction pressure vessel was charged with a mixture of tert-butyl (R)-2-(tert-butyldimethylsilyloxy)-3-(4-chloro-3-(4-chloro-6-(3,5-dimethylisoxazol-4-yl)-5-methylpyrimidin-2-yl)phenoxy)propyl (methyl)carbamate (480 mg, 0.74 mmol); 2-(azetidin-1-yl)-6,7-dihydro-5H-pyrrolo[3,4-d]pyrimidine 2,2,2-trifluoro acetate (0.77 mmol, 1.04 eq), DIPEA (0.8 mL). and DMSO (5 mL), the vessel was capped, placed in a microwave reactor and irradiated for 45 min. at external temperature of 110 °C. After being cooled down to room temperature, the mixture was diluted with EtOAc (100 mL) and washed with water (80 mL x 3). The organic phase was concentrated and the solid residue

purified on preparative tlc of silicagel developed with 1:2 petroleum ether:ethyl acetate to give tert-butyl (R)-(3-(3-(4-(2-(azetidin-1-yl)-5,7-dihydro-6H-pyrrolo[3,4-d]pyrimidin-6-yl)-6-(3,5-dimethyl isoxazol-4-yl)-5-methylpyrimidin-2-yl)-4-chlorophenoxy)-2-((tert-butyldimethylsilyl) oxy)propyl)(methyl)carbamate as a yellow solid (430 mg, 0.54 mmol, 73% yield). ESI-LCMS (m/z): 791.3 [M+H]+.

Step 3: Synthesis of (R)-1-(3-(4-(2-(azetidin-1-yl)-5,7-dihydro-6H-pyrrolo[3,4-d]pyrimidin-6-yl)-6-(3,5-dimethylisoxazol-4-yl)-5-methylpyrimidin-2-yl)-4-chlorophenoxy)-3-(methyl amino)propan-2-ol (Compound 4). A solution of tert-butyl (R)-(3-(3-(4-(2-(azetidin-1-yl)-5,7-dihydro-6H-pyrrolo[3,4-d]pyrimidin-6-yl)-6-(3,5-dimethylisoxazol-4-yl)-5-methyl pyrimidin-2-yl)-4-chlorophenoxy)-2-((tert-butyldimethylsilyl)oxy)propyl)(methyl)carbamate (430 mg, 0.54 mmol) in 90% aq. trifluoroacetic acid (4 mL) was stirred at room temperature for 60 min., the solvent was then removed in vacuo and the resulting residue was dissolved in MeOH (5 ml), treated with ammonia till pH 7-8 and concentrated. The crude material was purified by preparative HPLC to give (R)-1-(3-(4-(2-(azetidin-1-yl)-5,7-dihydro-6H-pyrrolo[3,4-d]pyrimidin-6-yl)-6-(3,5-dimethylisoxazol-4-yl)-5-methylpyrimidin-2-yl)-4-chlorophenoxy)-3-(methylamino)propan-2-ol as a white solid (100 mg, 0.17 mmol, 32% yield). ESI-LCMS (m/z): 577.1 [M+H]+. 1HNMR (400 MHz, CD3OD) δ ppm: 8.32 (s, 1H), 7.41 (d, J = 8.8 Hz, 1H), 7.32 (d, J =3.2 Hz, 1H), 7.05 (dd, J =3.2 and 8.8 Hz, 1H), 5.14 (s, 2H), 5.08 (s, 2H), 4.20-4.16 (m, 4H), 4.12-4.09 (m, 1H), 4.03-3.99 (m, 2H), 2.82-2.71 (m, 2H), 2.46 (s, 3H), 2.44 (m, 2H), 2.42 (s, 6H), 2.29 (s, 3H).

Synthesis of (*R*)-2-[2-[2-Chloro-5-(2-hydroxy-3-methylamino-propoxy)-phenyl]-6-(3,5-dimethyl-isoxazol-4-yl)-5-methyl-pyrimidin-4-yl]-2,7-diaza-spiro[3.5]nonane-7-carboxylic acid methyl ester (EZM2302)

Step 1: Synthesis of (R)-2-((3-bromophenoxy)methyl)oxirane. To a suspension of 3-bromophenol (100 g, 0.58 mol) and Cs_2CO_3 (379 g, 1.16 mol) in THF (1500 mL); *(R)*-oxiran-2-yl-methyl 3-nitrobenzenesulfonate (192 g, 0.74 mol) was added at room temperature. The reaction mixture was heated at 40 °C and stirred at the same temperature for 16h, cooled down to room temperature, filtered and concentrated. The residue was dissolved in water (200 mL) and extracted with ethyl acetate (150 mL x 3), the organic layers were combined, dried over Na_2SO_4, filtered and concentrated to give (*R*)-2-((3-bromophenoxy)methyl)oxirane (160 g, crude), which was used for the next step without further purification. ESI-LCMS (m/z): 228.7 $[M+1]^+$.

Step 2: Synthesis of (*R*)-1-(3-bromophenoxy)-3-(methylamino)propan-2-ol. A solution of (*R*)-2-((3-bromophenoxy)methyl)oxirane (159 g, crude from step 1) in MeOH (500 mL) stirred at 0 °C was treated with slow addition of 33% $MeNH_2$ in MeOH (500 mL). After the addition was complete the cooling bath was removed and the mixture was further stirred at room temperature for 16h; the volatiles were then removed in vacuo to give (*R*)-1-(3-bromophenoxy)-3-(methylamino)propan-2-ol (181 g, crude), which was used for the next step without further purification. ESI- LCMS (m/z): 260.0 $[M+1]^+$.

Step 3: Synthesis of (*R*)-tert-butyl 3-(3-bromophenoxy)-2-hydroxypropyl(methyl) carbamate. A solution of (*R*)-1-(3-bromophenoxy)-3-(methylamino)propan-2-ol (181 g, crude

from step 2) and triethylamine (178 g, 1.76 mol) in DCM (1 L) stirred at 0 °C was treated with portionwise addition of a solution of Boc_2O (289 g, 1.32 mol) in DCM (100 mL). After the addition was complete the cooling bath was removed and the reaction mixture was further stirred at room temperature for 2h., washed consecutively with water (300 mL x 2), aqueous NH_4Cl saturated solution (200 mL x 2) and brine (300 mL). The organic phase was dried over Na_2SO_4, filtered and concentrated to give (*R*)-tert-butyl 3-(3-bromophenoxy)-2-hydroxypropyl(methyl) carbamate (276 g, crude) as a pale yellow oil, which was used for the next step without further purification. ESI- LCMS (m/z): 382.0 $[M+23]^+$.

Step 4: Synthesis of (*R*)-tert-butyl 3-(3-bromophenoxy)-2-(tert-butyldimethyl-silyloxy)propyl(methyl)carbamate. A solution of (*R*)-tert-butyl 3-(3-bromophenoxy)-2-hydroxypropyl(methyl) carbamate (276 g, crude from step 3) and imidazole (132 g, 1.94 mol) in DCM (1 L) stirred at 0 °C under N_2 atmosphere, was treated with slow addition of TBSCl (189 g, 1.26 mol), the reaction mixture was further stirred at room temperature for 16h. and then successively washed with water (300 mL x 2) and brine (300 mL). The organic layer was dried over Na_2SO_4, filtered, concentrated and the resulting residue was purified by chromatographic column on silicagel (petroleum ether/EtOAc = 80/1 to 60/1) to give the (*R*)-tert-butyl 3-(3-bromo- phenoxy)-2-(tert-butyldimethylsilyloxy)propyl(methyl) carbamate (105 g, 39% yield for 4 steps) as a pale yellow oil. ESI-LCMS: 495.9 $[M+23]^+$.

Step 5: Synthesis of (*R*)-tert-butyl 2-(tert-butyldimethylsilyloxy)-3-(3-(4,6- dichloro-5-methylpyrimidin-2-yl)phenoxy)propyl(methyl)carbamate. A solution of (R)-tert-butyl 3-(3-bromophenoxy)-2-(tert-butyldimethylsilyloxy) propyl (methyl)carbamate (24 g, 50.58 mmol) in dry THF (100 mL), stirred at -78°C under N_2 atmosphere was treated with n-butyllithium (2.4 M in hexane, 21.1 mL) slowly added over 20 minutes. The mixture was stirred for another 10 minutes at the same temperature followed by slow addition of a solution of 4,6-dichloro-5-methyl- pyrimidine (9.1 g, 55.64 mmol) in THF (20 mL) and further stirred at -78°C for 30 minutes. DDQ (16.1 g, 70.9 mmol) was then added portion wise, the mixture warmed up to 0°C and stirred for 30 minutes at the same temperature, concentrated and the residue was diluted with CH_2Cl_2 (300 mL), successively washed with 10% NaOH (50 mL), water (100 mL × 2) and brine (100 mL). The organic layer was dried over Na_2SO_4, filtered, concentrated and the residue was purified by chromatographic column on silicagel eluted with petroleum ether/EtOAc = 80/1 to 40/1 to render (*R*)-tert-butyl 2-(tert-butyldimethylsilyloxy) -3-(3-(4,6-dichloro-5-methyl pyrimidin-2-yl)phenoxy)propyl(methyl) carbamate (10.8 g, 38% yield) as a white solid. ESI-LCMS (m/z): 578.2 $[M+23]^+$.

Step 6: Synthesis of tert-butyl (*R*)-2-(tert-butyldimethylsilyloxy) -3-(3-(4-chloro-6- (3,5-dimethylisoxazol-4-yl)-5-methylpyrimidin-2-yl)phenoxy) propyl(methyl) carbamate. To a solution of (*R*)-tert-butyl 2-(tert-butyldimethylsilyloxy)-3-(3-(4,6-dichloro- 5-methylpyrimidin-2-yl)phenoxy)propyl(methyl)carbamate (10.8 g, 19.4 mmol) in degassed dioxane and H_2O (3/1, 240 mL) was added 3,5-dimethylisoxazol-4-yl-boronic acid (2.73 g, 19.4 mmol), $Pd(PPh_3)_4$ (2.24 g, 1.94 mmol) and Na_2CO_3 (4.1 g, 38.81 mmol). The system was purged with N_2 stream and the mixture was stirred at 100 °C for 1 h., cooled down to room temperature, diluted with water (100 mL) and extracted with EtOAc (250 mL x 2). The organic layers were combined and washed with brine (200 mL), dried over Na_2SO_4, filtered, concentrated and the residue was purified by chromatographic column on silicagel, eluted with 0% to 15% EtOAc/ petroleum ether, to give

tert-butyl (*R*)-2-(tert-butyldimethylsilyloxy)-3-(3-(4-chloro-6-(3,5-dimethyl isoxazol-4-yl)-5-methylpyrimidin-2-yl)phenoxy)propyl (methyl)carbamate as a white solid (7.1 g, 59% yield). ESI-LCMS: 639.3 $[M+23]^+$.

Step 7: Synthesis of tert-butyl (*R*)-2-(tert-butyldimethylsilyloxy)-3-(4-chloro- 3-(4-chloro-6-(3,5-dimethylisoxazol-4-yl)-5-methylpyrimidin-2-yl)phenoxy) propyl(methyl)carbamate. To a solution of tert-butyl (R)-2-(tert-butyldimethylsilyloxy)-3-(3-(4-chloro-6-(3,5- dimethyl isoxazol-4-yl)-5-methylpyrimidin-2-yl)phenoxy)propyl(methyl)carbamate (7.1 g, 11.5 mmol) in DMF (100 mL) was added NCS (2.3 g, 17.25 mmol) and the reaction mixture was stirred at 25 ºC for 2 h., diluted with EtOAc (300 mL) and washed with water (200 mL x 3) followed by brine (200 mL). The organic layer was dried over Na_2SO_4, filtered and concentrated and the residue was purified by chromatographic column on silicagel, eluted with 0% to 15% EtOAc/ petroleum ether, to give tert-butyl (*R*)-2-(tert-butyldimethylsilyloxy)-3-(4-chloro-3-(4-chloro-6-(3,5-dimethylisoxazol-4-yl)-5-methylpyrimidin-2-yl)phenoxy)propyl(methyl)carbamate as white solid (6.2 g, 83% yield). ESI-LCMS: 673.2 $[M+23]^+$.

Step 7a: Synthesis of 2,7-diaza-spiro[3.5]nonane-2,7-dicarboxylic acid 2-tert- butyl ester 7-methyl ester. To a solution of 2,7-diaza-spiro[3.5]nonane-2-carboxylic acid tert-butyl ester (226 mg, 1.0 mmol) and triethylamine (303 mg, 3 mmol) in CH_2Cl_2 (6 mL) stirred at 0 °C under nitrogen atmosphere was added methyl chloroformate (188 mg, 2 mmol) dropwise, and the mixture was further stirred at room temperature for 1h., excess of reagent was quenched with saturated $NaHCO_3$ solution (10 mL) and the mixture was then extracted with EtOAc (20 mL x 2). The combined organic phase was washed with saturated NH_4Cl solution (20 mL) and brine (20 mL), dried over Na_2SO_4, filtered and concentrated to give 2,7-diaza-spiro[3.5]nonane- 2,7-dicarboxylic acid 2-tert-butyl ester 7-methyl ester as a white solid, which was used for the next step without further purification (assumed quantitative yield). ESI-LCMS (m/z): 307.2 $[M+23]^+$.

Step 7b: Synthesis of 2,7-Diaza-spiro[3.5]nonane-7-carboxylic acid methyl ester HCl salt
A solution of 2,7-diaza-spiro[3.5]nonane-2,7-dicarboxylic acid 2-tert-butyl ester 7-methyl ester (290 mg, 1.0 mmol, from step 7a) in MeOH (2 mL) was treated with 4N HCl in dioxane (6 mL), and the mixture was stirred at room temperature for 1 h., and then concentrated in vacuo to give 2,7-diaza-spiro[3.5]nonane-7-carboxylic acid methyl ester as HCl salt (530 mg, crude), which was used for the next step without further purification. ESI-LCMS (m/z): 185.2 $[M+H]^+$.

Step 8: Synthesis of (*R*)-2-[2-{5-[3-(tert-Butoxycarbonyl-methyl-amino)-2-(tert- butyl-dimethyl-silanyloxy)-propoxy]-2-chloro-phenyl}-6-(3,5-dimethyl-isoxazol-4-yl)-5-methyl-pyrimidin-4-yl]-2,7-diaza-spiro[3.5]nonane-7-carboxylic acid methyl ester. A reaction pressure vessel was charged with a mixture of tert-butyl (*R*)-2-(tert-butyldimethylsilyloxy)-3-(4-chloro-3-(4-chloro-6-(3,5-dimethylisoxazol-4-yl)-5-methylpyrimidin-2-yl)phenoxy)propyl (methyl)carbamate (120 mg, 0.18 mmol); 2,7-diaza-spiro[3.5]nonane -7-carboxylic acid methyl ester HCl salt (100 mg, crude from step 7b), triethylamine (93 mg, 0.92 mmol). and n-BuOH (3 mL), the vessel was capped, placed in a microwave reactor and irradiated for 60 min. at external temperature of 140 °C . After being cooled down to room temperature, the mixture was diluted with water (20 mL) and extracted with EtOAc (20 mL x 3). The organic layers were combined, dried over Na_2SO_4, filtered and concentrated to give (*R*)-2-[2-{5-[3- (tert-butoxycarbonyl-methyl-amino)-2-(tert-butyl-dimethyl-silanyloxy)-propoxy]-2-chloro-phenyl}-6-(3,5-dimethyl-

isoxazol-4-yl)-5-methyl-pyrimidin-4-yl]-2,7-diaza-spiro[3.5]nonane-7-carboxylic acid methyl ester as a light yellow solid (147 mg, crude) which was used for the next step without further purification. ESI-LCMS (m/z): 798.8 $[M+H]^+$.

Step 9: Synthesis of (*R*)-2-[2-[2-Chloro-5-(2-hydroxy-3-methylamino-propoxy)- phenyl]-6-(3,5-dimethylisoxazol-4-yl)-5-methyl-pyrimidin-4-yl]-2,7-diaza-spiro[3.5]nonane-7-carboxylic acid methyl ester (EZM2302). A solution of (R)-2-[2-{5-[3-(tert-butoxycarbonyl-methyl-amino) -2-(tert-butyl-dimethyl-silanyloxy)-propoxy]-2-chloro-phenyl}-6-(3,5-dimethyl-isoxazol-4-yl)-5-methyl- pyrimidin-4-yl]-2,7-diaza-spiro[3.5]nonane-7-carboxylic acid methyl ester (147 mg, from step 8) in 90% TFA (6.7 mL) was stirred at room temperature for 16 h., the solvent was removed in vacuo and the residue was dissolved in MeOH (5 ml), treated with ammonia till pH 7-8 and concentrated. The residue was purified by preparative HPLC to give (*R*)-2-[2-[2-Chloro -5-(2-hydroxy-3-methylamino-propoxy)-phenyl]-6-(3,5-dimethyl-isoxazol-4-yl)-5-methyl- pyrimidin-4-yl]-2,7-diaza-spiro[3.5]nonane-7-carboxylic acid methyl ester as a white solid (65 mg, 60% yield for 2 steps). ESI-LCMS (m/z): 585.3 $[M+H]^+$. ^{1}HNMR (500 MHz, CD_3OD) δ ppm: 7.42 (d, J = 8.5 Hz, 1H), 7.20 (d, J = 3.0 Hz, 1H), 7.06 (dd, J = 3.0 and 9.0 Hz, 1H), 4.36-4.25 (m, 1H), 4.19 (bs, 4H), 4.11-4.05 (m, 2H), 3.71 (s, 3H), 3.53-3.48 (m, 4H), 3.30-3.25 (m, 1H), 3.20-3.14 (m, 1H), 2.77 (s, 3H), 2.39 (s, 3H), 2.25 (s, 3H), 2.17 (s, 3H), 1.88-1.82 (m, 4H).

Synthesis of methyl 2-(2-(4-chloro-3-((*R*)-2-hydroxy-3-(methylamino)propoxy)phenyl)-6-(3, 5-dimethylisoxazol-4-yl)-5-methylpyrimidin-4-yl)-2,7-diazaspiro[3.5]nonane-7-carboxylate (EPZ029751)

Step 1: Synthesis of 4-bromo-1-chloro-2-(methoxymethoxy)benzene: A suspension of 5-bromo-2-chlorophenol (2.07 g, 10 mmol) and K_2CO_3 (30 mmol, 4.15 g) in DCM (20 mL), stirred at room temperature was treated with MOMBr (1.2 g, 15 mmol) and the resulting mixture was further stirred at the same temperature for 2 h., then quenched with H_2O (10 mL) and extracted with dichloromethane (2 x 50 mL). The combined organic phase was dried over Na_2SO_4, filtered and concentrated. The residue was purified by chromatographic column on silicagel eluted with 10% EtOAc in petroleum ether to give 4-bromo-1-chloro-2-(methoxymethoxy)benzene (2.49 g, 98% yield) as colorless oil. ^{1}H NMR (400 MHz, $CDCl_3$): δ 7.36 (d, J = 2.1 Hz, 1H), 7.25 (d, J = 8.5 Hz, 1H), 7.10 (dd, J = 2.2 and 8.5 Hz, 1H), 5.26 (s, 2H), 3.55 (s, 3H) ppm.

Step 2: Synthesis of 4, 6-dichloro-2-(4-chloro-3-(methoxymethoxy)phenyl)-5-methylpyrimidine: A solution of 4-bromo-1-chloro-2-(methoxymethoxy)benzene (1.0 g, 4.0 mmol,) in dry THF (10 mL), stirred at -78 ^oC under nitrogen atmosphere, was treated with dropwise addition of 2.5 M *n*-BuLi in hexanes (1.6 mL, 4 mmol ,) and the resulting mixture was further stirred at the same temperature for 0.5 h, then a solution of 4,6-dichloro-5-methylpyrimidine (652 mg, 4 mmol) in THF (2 mL) was slowly added and the reaction mixture was stirred at -78 ^oC for another 0.5 h. Subsequently, a solution of DDQ (908 mg, 4.0 mmol) in THF (2 mL) was added at -78 ^oC, the mixture was warmed to room temperature and stirred for another 0.5 h., diluted with water (20 mL) and extracted with dichloromethane (50 mL x 2). The combined organic phase was dried over Na_2SO_4, filtered, concentrated and the residue was purified by chromatographic column on silicagel eluted with 10% EtOAc in petroleum ether to give 4, 6-dichloro-2-(4-chloro-3-(methoxymethoxy) phenyl)-5-methylpyrimidine (432 mg,: 32%

yield) as white solid. ESI-LCMS (m/z): 333.2 $[M+1]^+$.

Step 3: Synthesis of 4-(6-chloro-2-(4-chloro-3-(methoxymethoxy)phenyl)-5-methyl-pyrimidin-4-yl)-3,5-dimethylisoxazole

$Pd(PPh_3)_4$ (116 mg, 0.1 mmol) was added to a suspension of 4,6-dichloro-2-(4-chloro-3-(methoxymethoxy)phenyl)-5-methyl-pyrimidine (333 mg, 1.0 mmol); 3,5-dimethyl isoxazol-4-ylboronic acid (141 mg, 1.0 mmol) and Na_2CO_3 (318 mg, 3.0 mmol) in degassed 5:1 dioxane:H_2O (60 mL) The system was purged with nitrogen stream and stirred at 100 ^oC for 2 h., cooled down to room temperature, diluted with water (100 mL), and the mixture was extracted with EtOAc (50 mL x 2). The combined organic phase was washed with brine, dried over Na_2SO_4, filtered and concentrated. The residue was purified by chromatographic column on silicagel eluted with 10% EtOAc in petroleum ether, to give 4-(6-chloro-2-(4-chloro-3-(methoxymethoxy)phenyl)-5-methylpyrimidin-4-yl)-3,5-dimethyl- isoxazole (182 mg, 46% yield) as white solid. ESI-LCMS: 394.0 $[M+1]^+$.

Step 4: Synthesis of methyl 2-(2-(4-chloro-3-(methoxymethoxy)phenyl)-6-(3,5-di-methylisoxazol-4-yl)-5-methylpyrimidin-4-yl)-2,7-diazaspiro[3.5]nonane-7-carboxylate: A reaction pressure vessel was charged with a mixture of 4-(6-chloro-2-(4-chloro-3-(methoxymethoxy)phenyl)-5-methyl- pyrimidin-4-yl)-3,5-dimethylisoxazole (315 mg, 0.8 mmol); methyl 2,7-diazaspiro[3.5]nonane-7-carboxylate HCl salt (262 mg, 1.2 mmol); DIPEA (1 mL) and DMSO (4 mL), the vessel was capped, placed in a microwave reactor and irradiated for 60 min. at external temperature of 140 °C . After being cooled down to room temperature, the mixture was diluted with water (20 mL) and extracted with EtOAc (50 mL x 3). The organic layers were combined, dried over Na_2SO_4, filtered and concentrated to give 2-(2-(4-chloro-3-(methoxymethoxy)phenyl)-6-(3,5-di-methylisoxazol-4-yl)-5-methylpyrimidin-4-yl)-2,7-diazaspiro[3.5]nonane-7-carboxylate (433 mg, crude), which was submitted to the next step without further purification. ESI-LCMS (m/z): 542.2$[M+H]^+$.

Step 5: Synthesis of methyl 2-(2-(4-chloro-3-hydroxyphenyl)-6-(3,5-dimethyl- isoxazol-4-yl)-5-methylpyrimidin-4-yl)-2,7-diazaspiro[3.5]nonane-7-carboxylate: A solution of 2-(2-(4-chloro-3-(methoxymethoxy)phenyl)-6-(3,5-dimethylisoxazol-4-yl)-5-methylpyrimidin-4-yl)-2,7-diazaspiro[3.5]nonane-7-carboxylate (433 mg, crude from step 4) in 50% TFA in DCM (10 mL) was stirred at room temperature for 2 h. After the reaction was complete, the solution was concentrated, the residue was suspended in water (20 mL) and the pH adjusted to 7-8 with saturated aqueous Na_2CO_3 solution. The mixture was then extracted with EtOAc (50 mL x 2), the combined organic phase was dried over Na_2SO_4, filtered and concentrated to give methyl 2-(2-(4-chloro-3-hydroxyphenyl)-6-(3,5-dimethyl-isoxazol-4-yl)-5-methylpyrimidin-4-yl)-2,7-diazaspiro[3.5]nonane-7-carboxylate (400 mg, crude), which was submitted to the next reaction without further purification. ESI-LCMS (m/z): 498.1 $[M+1]^+$.

Step 6: Synthesis of methyl 2-(2-(4-chloro-3-((*R*)-oxiran-2-ylmethoxy)phenyl)-6-(3,5-dimethylisoxazol-4-yl)-5-methylpyrimidin-4-yl)-2,7-diazaspiro[3.5]nonane-7-carboxylate: A suspension of methyl 2-(2-(4-chloro-3-hydroxyphenyl)-6-(3,5-dimethylisoxazol-4-yl)-5-methylpyrimidin-4-yl)-2,7-diazaspiro[3.5]nonane-7-carboxylate (400 mg, crude from step 5) and Cs_2CO_3 (652 mg, 2 mmol) in THF (10 mL) was treated with (*R*)-oxiran-2-yl-methyl 3-nitrobenzenesulfonate (259 mg, 1.3 mmol) and the mixture stirred at 40 ^oC overnight; cooled

down to room temperature, diluted with water (20 mL) and extracted with EtOAc (100 mL x 2). The combined organic phase was dried over Na_2SO_4, filtered and concentrated to give methyl 2-(2-(4-chloro-3-((*R*)-oxiran-2-ylmethoxy)phenyl)-6-(3,5-dimethylisoxazol-4-yl)-5-methylpyrimidin-4-yl)-2,7-diazaspiro[3.5]nonane-7-carboxylate (150 mg, crude), which was used for next step without further purification. ESI-LCMS (m/z): 554.0 $[M+1]^+$.

Step 7: Synthesis of methyl 2-(2-(4-chloro-3-((*R*)-2-hydroxy-3-(methylamino)propoxy) phenyl) -6-(3,5-dimethylisoxazol-4-yl)-5-methylpyrimidin-4-yl)-2,7-diazaspiro[3.5]nonane-7-carboxylate (Compound 4): A solution of methyl 2-(2-(4-chloro-3-((*R*)-oxiran-2-ylmethoxy)phenyl)-6-(3,5-dimethylisoxazol-4-yl)-5-methyl-pyrimidin-4-yl)-2,7-diazaspiro[3.5]nonane-7-carboxylate (150 mg, crude from step 6) in 33% methylamine in ethanol (5 mL) was stirred at room temperature for 2 h, the volatiles removed in vacuo and the residue was purified by HPLC to render methyl 2-(2-(4-chloro-3-((*R*)-2-hydroxy-3-(methylamino) propoxy)phenyl)-6-(3,5-dimethylisoxazol-4-yl)-5-methylpyrimidin-4-yl)-2,7-diazaspiro [3.5]nonane-7-carboxylate (63 mg, 13% yield for four steps) as white solid. ESI-LCMS (m/z): 585.0 $[M+1]^+$; ^1H NMR (400 MHz, CD_3OD) δ ppm: 8.03 (d, J = 1.9 Hz, 1H), 7.96 (dd, J = 1.8 and 8.4 Hz, 1H), 7.45 (d, J = 8.3 Hz, 1H), 4.29-4.09 (m, 7H), 3.70 (s, 3H), 3.55-3.49 (m, 4H), 3.18-3.01 (m, 2H), 2.64 (s, 3H), 2.38 (s, 3H), 2.26 (s, 3H), 2.13 (s, 3H), 1.89-1.80 (m, 4H) ppm.

Protein production:

FLAG-CARM1 (amino acids 2-585) -His (amino acids 2-585) for biochemical studies was expressed in 293F mammalian cells using standard methodologies. Cells were lysed by sonication in a buffer containing 20 mM Tris, 150 mM NaCl, 5% glycerol, pH 7.8 with Roche protease inhibitor cocktail dissolved and then centrifuged to remove cell debris. Supernatant was loaded onto Anti-FLAG M2 affinity gel column (Sigma) pre-equilibrated with buffer containing 20 mM Tris, 500 mM NaCl, 5% glycerol, pH 7.8 and eluted with 20 mM Tris, 150 mM NaCl, 5% glycerol, pH 7.8, 200 μg/ml FLAG peptide. After dialysis into 20 mM Tris, 150 mM NaCl, 5% glycerol, 1 mM DTT pH 7.8, CARM1 was concentrated to at least 1 mg/ml prior to flash freezing.

Protein for protein crystallography was produced using insect cells. His-TEV-CARM1 (amino acids 134-479) was expressed in Hi Five cells using standard procedures for baculovirus expression. Cell pellets were resuspended in 50 mM Tris, 300 mM NaCl, 10% glycerol, pH 7.8 with Roche protease inhibitor cocktail added and were lysed by sonication. Lysate was centrifuged to remove cell debris. Ni affinity resin was pre-incubated with lysis buffer before incubation with the supernatant. Resin was sequentially washed with lysis buffer containing increasing amounts of imidazole (up to 50 mM) before elution with 250 mM imidazole. After digestion with TEV protease, the protein was dialyzed into lysis buffer to remove imidazole. The protein was passed over a nickel affinity column to remove any uncleaved protein. The protein was then purified by gel filtration using an S200 column pre-incubated with 20 mM Tris, 50 mM NaCl, 1 mM βME, pH 7.2. Protein was concentrated to >15 mg/ml, aliquoted, frozen in liquid nitrogen and stored at -80 °C until needed for crystallization.

X-ray crystallography

SAM was solubilized at 100 mM in DMSO before addition to CARM1 protein (2 mg/ml) at a final concentration of 0.5 mM. Vapor diffusion methods utilizing hanging drop trays with a 0.5

mL reservoir were used for crystallization. Typically, 2 μL protein was added to 1 μL well solution containing 0.2 M Ammonium Sulfate, 0.1 M Tris pH 8.5, 18% w/v PEG 3350. Trays were incubated at 18º C. After solubilization at 100 mM in DMSO, compounds were soaked into the crystals using buffer containing 0.2 M Ammonium Sulfate, 0.1 M Tris pH 8.5, 18% w/v PEG 3350, and 1 mM compound (1% v/v DMSO). After 12-24 hours, crystals were cryoprotected in a solution containing 80% mother liquor and 20% glycerol prior to freezing in liquid nitrogen. All datasets were collected at synchrotron sources at -180°C. Data reduction was performed using XDS [1] and scaling was performed using either SCALA [2] (Compound **2**) or AIMLESS [3] (EZM2302). Structure determination was performed using previously solved structures of CARM1 and visual inspection of difference density maps. Dictionaries were generated using ProDrg [4] within the CCP4 software package and ligand fitting of SAH and compound were performed manually. Structure refinement was performed using iterative cycles of refinement and model building using REFMAC5 [5] and COOT [6] , respectively. Analysis of the structures show greater than 99% of all residues are in preferred or allowed regions of the Ramachandran plot. Data collection and refinement statistics are shown in Supplementary Table 1.

Six-day cell proliferation assay

Optimal cell seeding density was determined empirically for each cell lines by examining the growth of a wide range of seeding densities in a 384-well format to identify conditions that permitted maximal and continued proliferation for 6 days. Cells were plated in duplicate plates 24 hours before treatment with a 20-point two-fold dilution series of compound or 0.15% DMSO. Plates were incubated for 6 days at 37°C in 5% CO2. Cells were then lysed with CellTiter-Glo (CTG) (Promega) and chemiluminescent signal was detected with an appropriate microplate reader. In addition, an untreated plate of cells was harvested at the time of compound addition (T0) to quantify the initial cell density. CTG values obtained after 6 days of treatment were expressed as a percent of the T0 value and plotted against compound concentration. Data were fit with a four parameter equation to generate a concentration response curve from which growth IC_{50} values were calculated.

SmB antibody validation

HCT-116 cells (ATCC) were transiently transfected with Silencer Select siRNAs (Invitrogen) against SmB or a non-targeting control (NTC) siRNA, using RNAiMAX transfection reagent (Invitrogen). Cells were pelleted 72 hours after transfection and lysates were analyzed by Western blotting.

A375 cells (ATCC) were transduced with CARM1 or NTC shRNAs (Sigma-Aldrich) in a TRIPZ inducible vector and selected and maintained in 1 µg/mL puromycin. CARM1 shRNA expression was induced with 1ug/mL doxycycline. Pellets were collected after 15 days of doxycycline induction and lysates were analyzed by Western blotting.

Unmethylated or dimethylated SmB peptides (21st Century Biochemicals) were spotted on nitrocellulose membranes at 10 to 0.005 ng, and membranes were blocked and blotted as for Western Blotting with SmB primary antibody (Sigma-Aldrich, 12F5, S0698). After incubation, membrane was developed as for a Western blot. The amino acid sequences of the peptides were: Peptide 1: C-Ahx-GMMGPPPGM[Rme2a]PPMGPPMGIPPG[Rme2a]GTPMGMP-amide;

Peptide 2: C-Ahx-PMGMPPPGM[Rme2a]PPPPGM[Rme2a]GPPPPGM[Rme2a]PP[Rme2a]P-OH.

Histone methyltransferase assay

Compound IC_{50} values were determined by performing a 10 point 3-fold serial dilution in duplicate with 1 mM SAH and DMSO as minimum and maximum signals controls respectively. The compounds were preincubated with CARM1 for 30 minutes at room temperature before the reactions were initiated by the addition of substrates. Final assay conditions were 0.25 nM CARM1, 30 nM ^{3}H-S-adenosyl-methionine (SAM), and 250 nM biotinylated peptide in buffer containing 20 mM bicine, 1 mM tris(2-carboxyethyl)phosphine, 0.005% bovine skin gelatin and 0.002% Tween-20, pH 7.5 and 2% DMSO. The peptide sequence was biotin-aminohexanoate-PRKQLATKAARMeKSAP-amide, where RMe is a monomethylated arginine residue (21st Century Biochemicals, Marlboro, MA). After 2 hours, assays were quenched by the addition of 300 µM unlabeled SAM. The reactions were transferred to a 384-well Flashplate (Perkin Elmer) and the biotinylated peptides were allowed to bind to the streptavidin surface for at least 1 hour before being washed once with 0.1% Tween 20. The quantity of ^{3}H-labeled peptide bound to the Flashplate surface was counted on a Topcount reader as counts per minute (cpm). % Inhibition values were calculated and IC_{50} values were fit using the 3-parameter IC_{50} equation below:

$$Y = Bottom + \frac{(100 - Bottom)}{(1 + \left(\frac{X}{\mathrm{IC}_{50}}\right)^{Hill\,Coefficient})}$$

where top is fixed at 100 or the maximum inhibition plateau, Y is % inhibition and X is compound concentration.

Substrate competition studies were performed by measuring compound IC_{50} values at varying peptide (4000, 2000, 1000, 500, 250, 125, 62.5, 31.3 nM) and SAM concentrations (400, 200, 100, 50, 25, 12.5, 6.25 nM). IC_{50} *vs* [peptide] data were fit to the noncompetitive Cheng-Prusoff equation [7].

The effect of reaction time on compound potency was determined by measuring IC_{50} values from reactions quenched at 10, 20, 40, 60, 90, 120, 150, 180 mins. IC_{50} *vs* reaction time data were fit to a one phase exponential decay equation.

For the dual inhibitor study, EZM2302 (final concentrations = 20, 10, 5, 2.5, 1.25, 0.63, 0.31 and 0 nM) and SAH (final concentrations = 30, 15, 7.5, 3.8, 1.9, 0.94, 0.47 and 0 nM) were titrated in duplicate in a matrix, where the final DMSO concentration was normalized to 2%. CARM1 activity was measured under standard conditions after a 30 minute preincubation. EZM2302 IC_{50} values were determined and data were analyzed using the following equation:

$$\mathrm{IC}_{50}^{I} = K_I \left(\frac{1 + \frac{J}{K_J}}{1 + \frac{J}{\propto K_J}} \right)$$

where K_I and K_J are the inhibition constants for I and J, respectively, and alpha is a constant describing the effect of one inhibitor on the binding of another inhibitor [8].

Reversibility experiments were performed in assay buffer lacking bovine skin gelatin. CARM1 (1.2 μM) was incubated with DMSO (2.5%) or 10 μM EZM2302 in the presence or absence of 100 μM SAH, or 50 μM SAM and 300 uM peptide at room temperature for 2 hours. The mixture was purified using pre-equilibrated 0.5 mL Zeba (7MWCO) spin columns (Thermo Fisher) following manufacturer's instructions. CARM1 was diluted to 0.5 nM in an assay reaction mixture using previously described conditions and activity was measured over 2 hours. Proteins were kept at 4°C for further activity testing. Similar results were also obtained when CARM1 was incubated with EZM2302 under more dilute conditions (0.63 nM CARM1, 75 nM EZM2302 and 750 nM SAH) and purified with 5 mL Zeba (40K MWCO) spin columns.

Methyltransferase cross screening panel

Cross screening against the protein methyltransferase enzymes listed in Figure 1B followed the general procedures from Knutson et al.[9]

In cell western assay for detection of asymmetric di-methyl PABP1 and DNA content

RKO cells (ATCC) were maintained in growth medium (DMEM/Glutamax medium supplemented with 10% v/v heat inactivated fetal bovine serum and 100 units/mL penicillin-streptomycin) and cultured at 37 °C under 5% CO2. Cells were seeded in assay medium at a concentration of 30,000 cells per mL to a poly-D-lysine coated 384 well culture plate with 50 µL per well. Compound (100 nL) from a 96-well source plate was added directly to 384 well cell plate. Plates were incubated at 37°C, 5% CO2 for 48 hours. After two days of incubation, plates were brought to room temperature outside of the incubator for ten minutes and blotted on paper towels to remove cell media. Cells were fixed for 20 minutes at room temperature by adding 50 µl of 8% PFA followed by aspiration of supernatant. Cells were then permeabilized by addition of 50 µL of ice cold 100% methanol directly to each well and incubated for 30 min at room temperature. After 30 min, plates were washed 2 times with 100 µL per well of wash buffer (1X PBS). Next 60 µL per well of Odyssey blocking buffer (Odyssey Buffer with 0.1% Tween 20 (v/v)) were added to each plate and incubated 1 hour at room temperature. Blocking buffer was removed and 20 µL per well of primary antibody was added (asymmetric-methyl PABP1) diluted 1:400 in Odyssey buffer with 0.1% Tween 20 (v/v)) and plates were incubated overnight (16 hours) at 4°C. Plates were washed 5 times with 100 µL per well of wash buffer. Next 20 µL per well of secondary antibody was added (1:800 800CW goat anti-rabbit IgG (H+L) antibody, 1:2000 DRAQ5 in Odyssey buffer with 0.1% Tween 20 (v/v)) and incubated for 1 hour at room temperature. The plates were washed 5 times with 100 µL per well wash buffer then 2 times with 100 µL per well of water. Plates were allowed to dry at room temperature then imaged on the Licor Odyssey machine. The ratio of asymmetric di-methyl PABP1 to DRAQ5 was determined for each well and then used to determine percent inhibition relative to DMSO control. IC_{50} curves were generated using triplicate wells per concentration of compound.

Hepatocyte stability

Compounds (1 µM; 0.25% DMSO final) were added to 96 well plates containing 0.25 x 10^6 pooled cryopreserved mixed human hepatocytes in 0.5 mL of Williams E media to start the

reaction. Plates were incubated at 37°C and 50 µL aliquots were removed from the incubation mixture at 0, 10, 20, 40, 60 and 90 min and added to 2 volumes of methanol containing metoprolol (internal standard) to stop the reaction. Negative control incubations with inactivated cell lysate were incubated for 90 min. Positive controls were verapamil and umbelliferone. The termination plates were centrifuged at 2,500 rpm for 20 min at 4°C to precipitate the protein prior to LC-MS/MS analysis. *In vitro* half-life ($t_{½}$) value was determined by plotting the natural logarithm of the analyte/IS peak area ratios as a function of time, with the slope of the linear regression (k; - gradient) converted to *in vitro* $t_{½}$ value where $t_{½}$=0.693/k. Subsequently, intrinsic CL (CL_{int}) was calculated as: (incubation volume/number of cells)x0.693/$t_{½}$ and scaled CL values were obtained using the well-stirred venous equilibration model.

Plasma Protein Binding

Plasma protein binding was assessed by equilibrium dialysis, utilizing the HT-dialysis cell format with a cellulose semi-permeable membrane (molecular weight cut-off of 5000 Da). Solutions of EZM2302 (5 µM, 0.5% final DMSO) were prepared in isotonic phosphate buffer (pH 7.4) and 100% mixed gender pooled human, pooled male CD-1 mouse or Sprague-Dawley rat plasma. The buffer solution was added to one side of the membrane and the plasma solution to the other side. Incubations were performed in duplicate for 16 h, at 37°C, in order to allow the compounds to reach equilibrium. Haloperidol was incubated in parallel as the control compound for each species. At the end of the incubation time, samples were taken from both sides of the membrane. Following protein precipitation, the samples were analyzed by LC-MS/MS using two sets of calibration standards for protein free (7 points) and protein containing solutions (6 points). Samples were quantified using standard curves prepared in the equivalent matrix. The fraction unbound in plasma (f_u) was calculated using the following equation:

$$f_u^{corrected} = \frac{PF}{\{(PC - PF) \times V_{Correction}\} + PF}$$

Where, PC = sample concentration in protein containing side; PF = sample concentration in protein free side and $V_{Correction}$ = correction factor for the volume shift i.e. ratio of the volume of the protein after dialysis to that before dialysis.

Cytochrome P450 Inhibition (IC_{50} Determination)

EZM2302 at 0, 0.1, 0.25, 1, 2.5, 10, 25 µM (final DMSO concentration 0.3%) was incubated with human liver microsomes (0.1 to 1 mg/mL) and NADPH (1 mM) in the presence of specific probe substrates at 37°C. Probe substrates and incubation times were: phenacetin (30 µM, 5 min), tolbutamide (120 µM, 60 min), mephenytoin (25 µM, 60 min) and dextromethorphan (5 µM, 5 min) for CYP1A2, CYP2C9, CYP2C19 and CYP2D6, respectively. For CYP3A4, midazolam (2.5 µM, 5 min) and testosterone (50 µM, 5 min) were used as probe substrates. Selective P450 inhibitors were screened alongside EZM2302 as positive controls for each isoform. All reactions were terminated by addition of methanol prior to centrifugation and addition of internal standard to the supernatants. For CYP2C9, CYP2C19, CYP2D6 and CYP3A4 (midazolam) incubations, the supernatants were combined for the simultaneous analysis of 4-hydroxytolbutamide, 4-hydroxymephenytoin, dextrorphan and 1-hydroxymidazolam by LC-MS/MS. For the CYP1A2 and CYP3A4 (testosterone) incubations, the supernatants were analyzed individually for acetaminophen and 6ß-hydroxytestosterone by LC-MS/MS. A decrease in the formation of the metabolites compared to vehicle control was used to calculate an IC_{50} value.

Supplementary Table S1: Crystallographic data collection and refinement statistics for inhibitor complexes in CARM1

Compound	**2**	**EZM2302**
PDB code	6ARV	6ARJ
Space group	$P2_12_12$	$P2_12_12$
	75.2, 99.1, 208.9	75.2, 98.8, 208.3
	90.0, 90.0, 90.0	90.0, 90.0, 90.0
Resolution range (Å)	78.78-2.00	48.07-1.92
(Highest resolution shell)	(2.05-2.00)	(1.96-1.92)
Rmerge $_{\text{overall}}$ [1]	0.086 (0.761)	0.076 (0.713)
Completeness overall (%)	99.2 (98.8)	99.8 (97.2)
Reflections, unique	105501	118082
Multiplicity	4.7 (4.4)	7.3 (7.1)
I/σ	11.9 (2.3)	13.3 (2.0)
Rvalue $_{\text{overall}}$ (%) [2]	19.1	17.5
Rvalue $_{\text{free}}$ (%)	23.2	21.2
R.m.s. deviations from ideal values		
Bond lengths (Å)	0.010	0.010
Bond angles (º)	1.348	1.432
Φ, Ψ angle distribution for residues [3]		
In preferred regions (%)	96.6	96.9
In generously regions (%)	3.1	2.6
In disallowed regions (%)	0.4	0.5

[1] $R_{\text{merge}} = \Sigma_{hkl} [(\Sigma_i |I_i - \langle I \rangle|)/ \Sigma_i I_i]$

[2] $R_{\text{value}} = \Sigma_{hkl} ||F_{\text{obs}}| - |F_{\text{calc}}|| / \Sigma_{hkl} |F_{\text{obs}}|$

R_{free} is the cross-validation R factor computed for the test set of 5 % of unique reflections

Supplementary Table S2. Long term proliferation IC_{50} values of cell lines dosed with EZM2302. A panel of cell lines was treated with EZM2302 for 15 days in a long term proliferation assay. Absolute IC_{50} values were calculated in GraphPad Prism (non-linear regression analysis, top of the curves were fixed to 100%) for each line at day 15.

Cell line	Cancer Primary Site	EZM2302 IC50 (µM)
ZR751	BREAST	>20
BT20	BREAST	>20
BT474	BREAST	>20
MCF7	BREAST	>20
DLD1	LARGE	>20
HCT116	LARGE	>20
HT29	LARGE	>20
RKO	LARGE	>20
DU145	PROSTATE	>20
LNCAP	PROSTATE	12.2
PC3	PROSTATE	>20
VCAP	PROSTATE	>20

Supplementary Table S3. Proliferation IC_{50} values of cell lines dosed with EZM2302. A panel of cell lines was treated with EZM2302 for 6 days in a proliferation assay. Cell density (as assessed by CellTiter-Glo) at day 0 was compared to day 6 to determine growth IC_{50} values, calculated using a four parameter dose response equation.

Cell Type According to Supplier	Cell Type Category	Cell Line Name	gIC_{50} Modifier	Average day 6 gIC_{50} (nM)	Standard Deviation	Replicates
MM	Plasma cell neoplasm	MM.1R		216	147	6
MM		NCI-H929		862	2034	10
MM		LP-1		2645	2699	6
MM		OPM-2		4952	7627	6
MM		MOLP-8		6623	2389	3
Plasma cell leukemia		JJN3	>	12954	7389	7
MM (Bone marrow)		KMS-12BM	>	15479	17783	8
Plasma cell leukemia		L363		16075	5470	7
MM (Pleural effusion)		KMS-12PE	>	17905	14854	5
Myeloma		KMS-11	>	18152	10918	12
MM		EJM	>	20189	15700	3
Plasma cell leukemia		SK-MM-2		20447	3886	4
MM		U266B1		22120	8100	8
Plasmacytoma		AMO-1	>	25929	14571	3
MM		RPMI-8226	>	26144	11578	8
MM		MOLP-2	>	30679	10215	6
AML	AML	MOLM-16		6535	6561	4
AML		MV-4-11	>	7859	5729	5
AML		KASUMI-1		13331	244	2
AML		GDM-1		20698	1899	2
AML M6; Erythroleukemia		TF-1		25938	12089	2
AML		UT-7	>	27626	15643	3
AML		SHI-1	>	36037	1074	3
AML		NOMO-1	>	36547	191	3
AML		SH-2	>	36657	0	2
AML		UCSD-AML1	>	36657	0	2
tFL	General B-cell lymphoma	DOHH-2		468	76	2
B-cell lymphoma		JM1	>	7331	0	2
B-NHL		REC-1	>	7331	0	2
Burkitt lymphoma		NC-37	>	7331	0	2
GCB DLBCL		DB	>	7331	0	2
GCB DLBCL		HT	>	7331	0	2
EBV+ B-lymphoblastoid	Normal	MC/CAR		241	123	9
EBV+lymphoblastoid		ARH-77	>	4266	11410	10
Transformed B-lymphoblastoid (GM4672 treated with EMS)		HuNS1	>	19027	15958	8

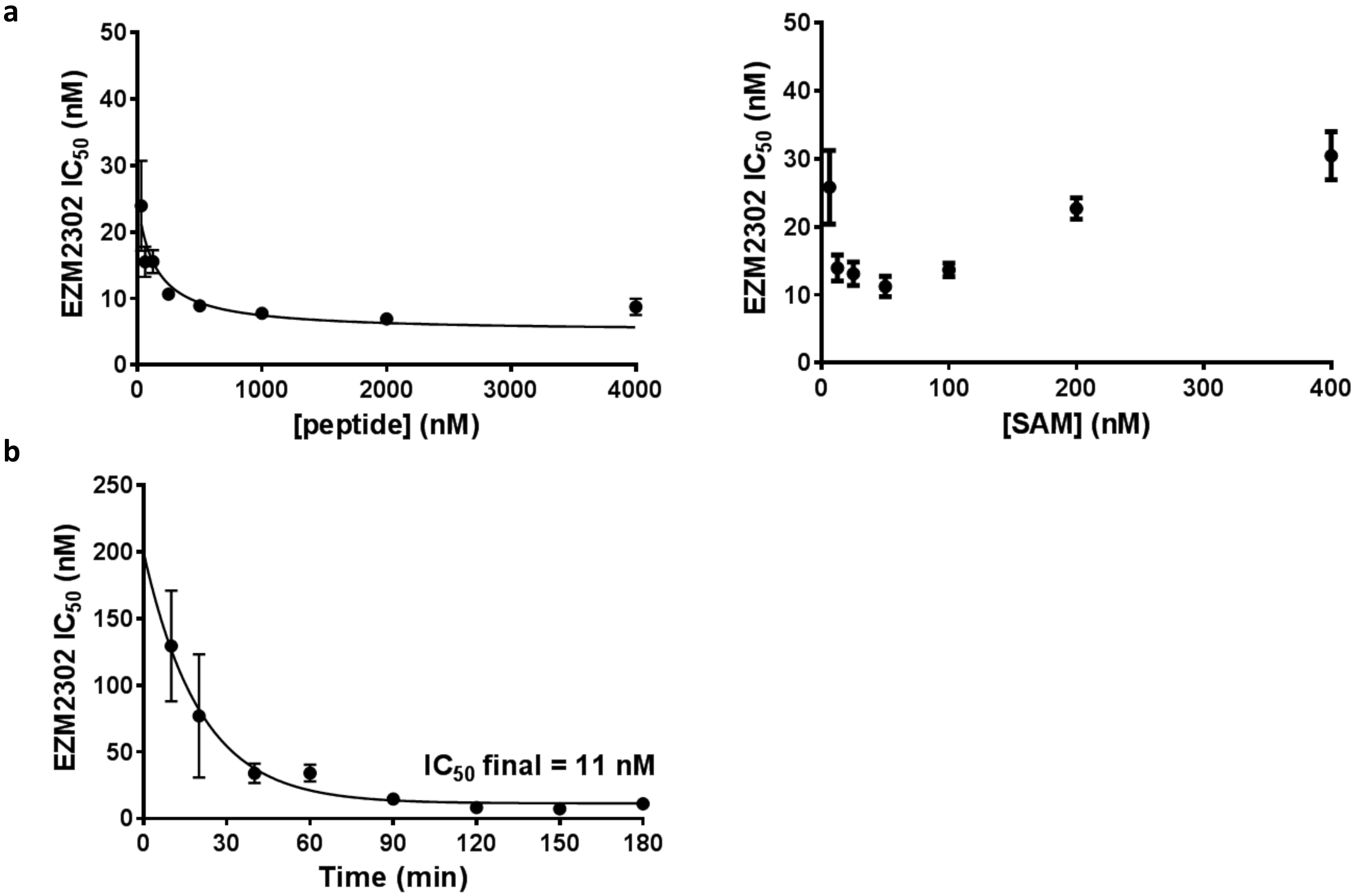

Supplementary Figure S1. **EZM2302 mechanism of inhibition studies. A,** Substrate competition studies with EZM2302. IC_{50} values were determined at various concentrations of peptide or SAM. Data fit best to a noncompetitive inhibition model, where K_i = 25 ± 3 nM and α = 0.2. **B,** Time-dependent inhibition of CARM1 by EZM2302. IC_{50} values for EZM2302 decrease with increasing reaction time.

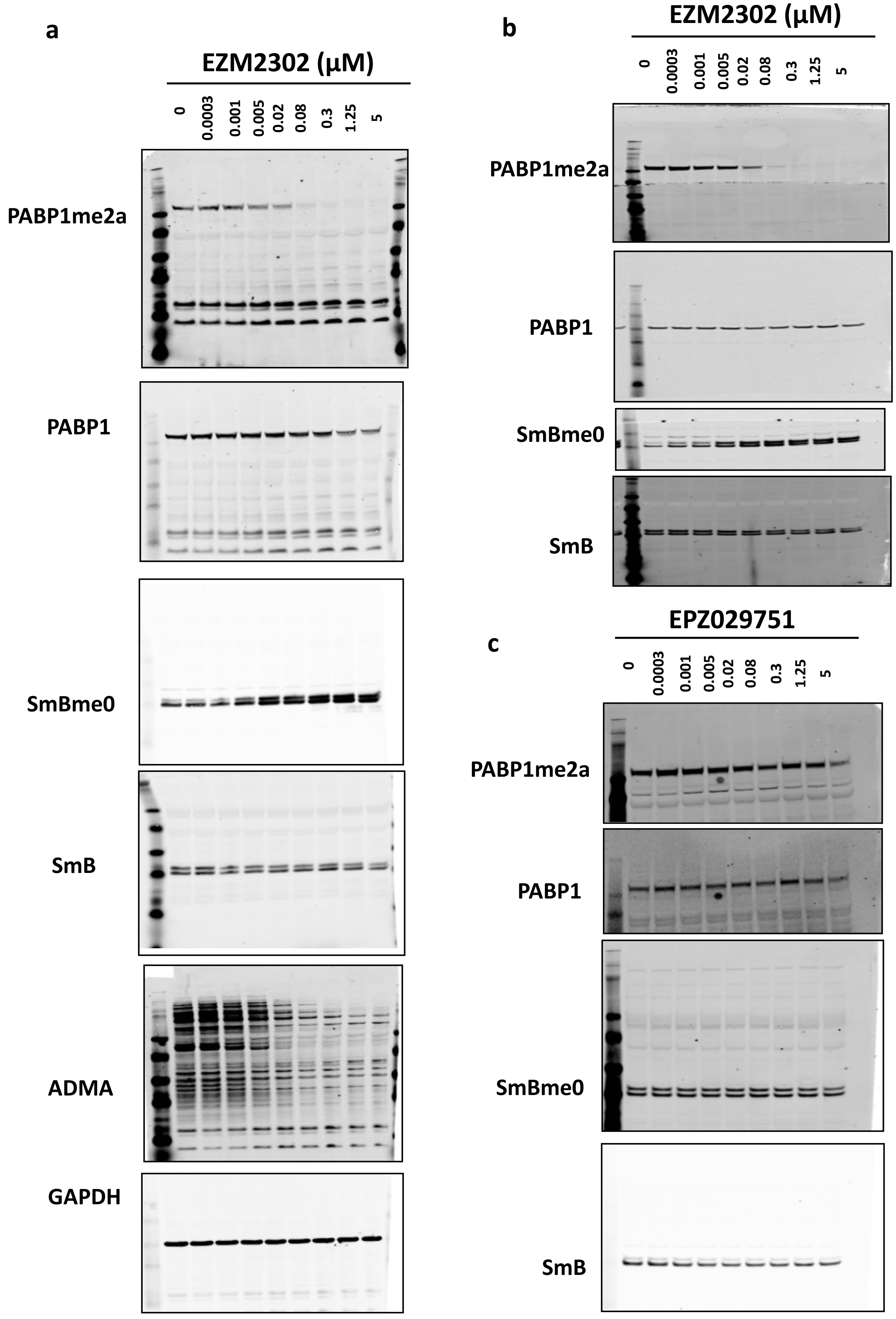

Supplementary Figure S2. Uncropped images from Figure 3. A. Figure3A **B.** Figure 3B. **C.** Figure 3C.

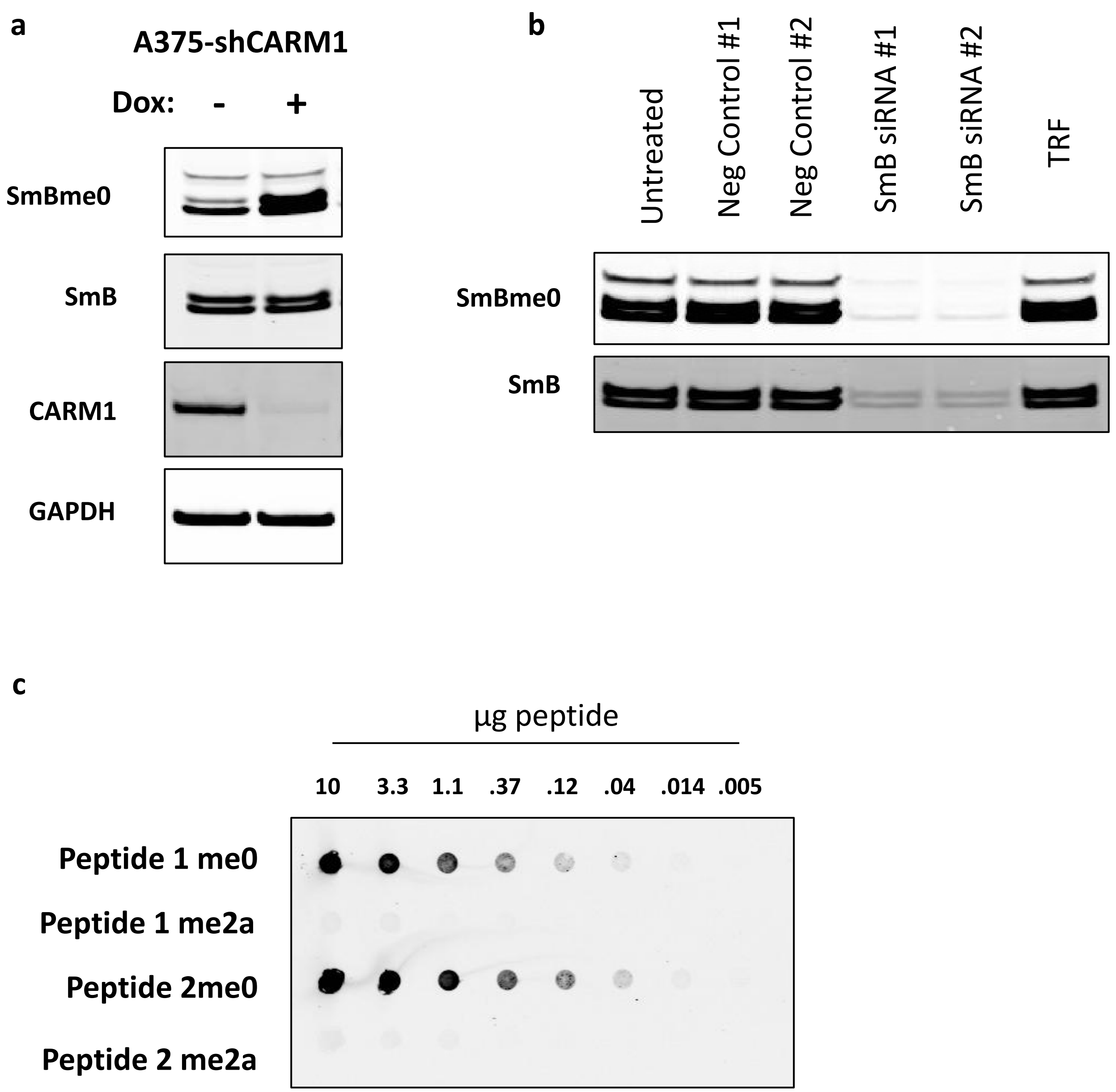

Supplementary Figure S3. SmBme0 antibody selectively detects unmethylated SmB with two transcript variants, SmB and SmB', detected. A, Knockdown of CARM1 for 15 days in A375 cells with doxycycline-inducible shRNA (1µg/mL doxycycline) increases levels of the unmethylated form of SmB without impacting levels of total SmB protein. B, Treatment of HCT-116 cells with SmB siRNA for 72 hours results in loss SmB protein as detected by both the SmB and SmBme0 antibodies. C, SmBme0 antibody detects unmethylated, but not asymmetrically dimethylated SmB peptides. Images in **A** and **B** are cropped. Uncropped images are in Supplementary Fig. S4.

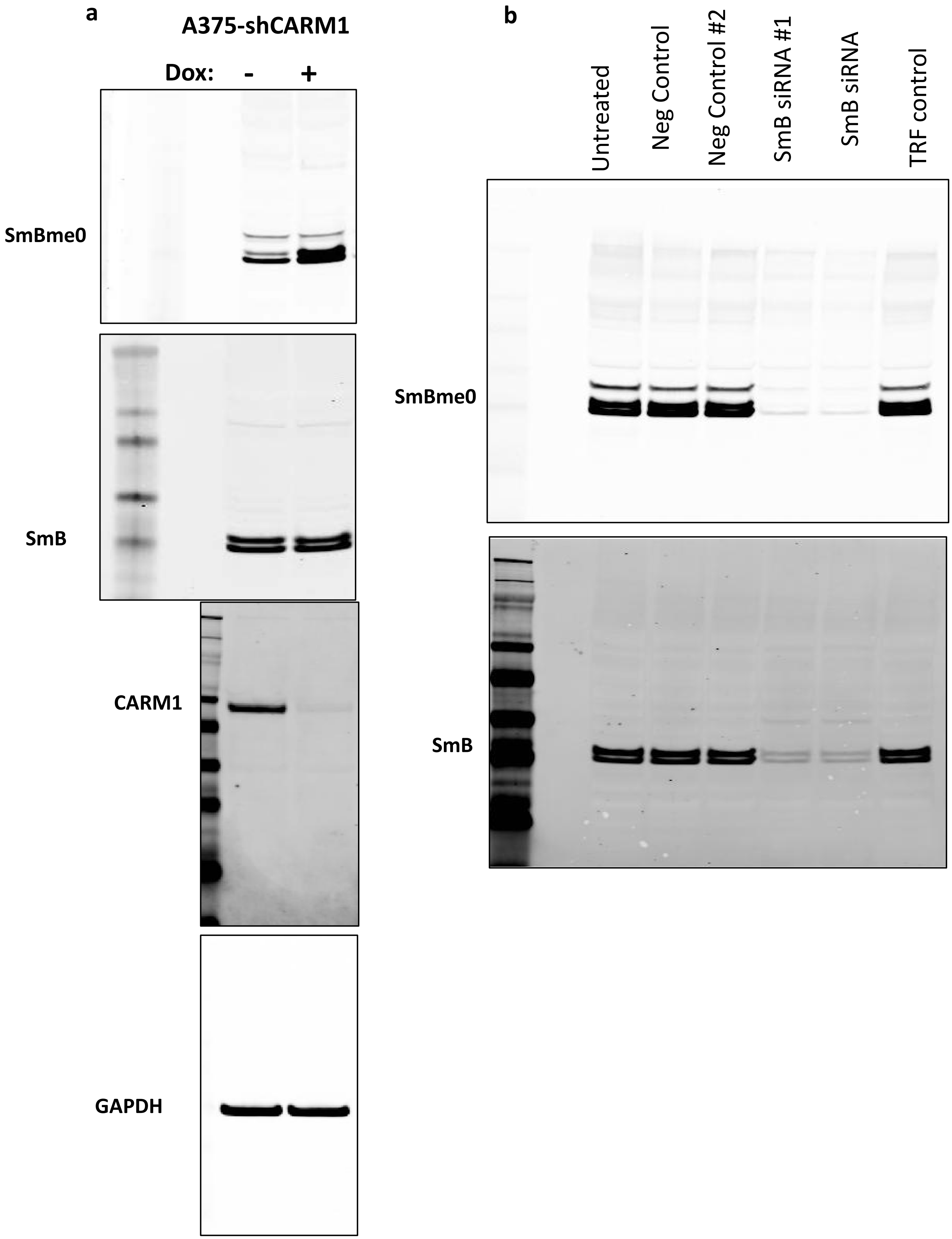

Supplementary Figure S4. Uncropped images from Supplementary Figure S3.

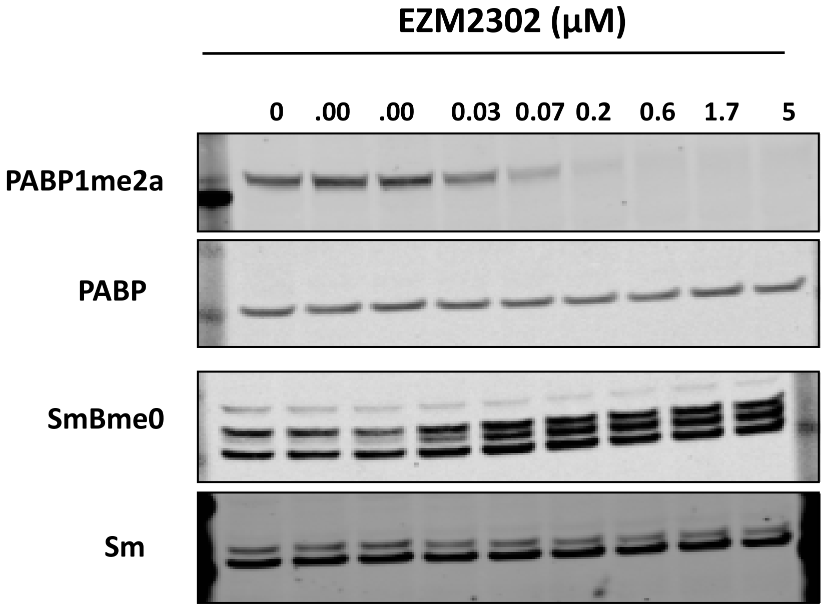

Supplementary Figure S5. Effects of EZM2302 on cellular target inhibition using western blot. Concentration-dependent inhibition of cellular asymmetric dimethyl arginine substrates, after four days of EZM2302 treatment in the U266B1 multiple myeloma cell line. Cells were treated with a dose-titration of 0.0003 to 5 µM compound. U266B1 IC_{50} values were calculated as 0.022 µM (PABP1me2a), 0.020 µM (SmBme0).

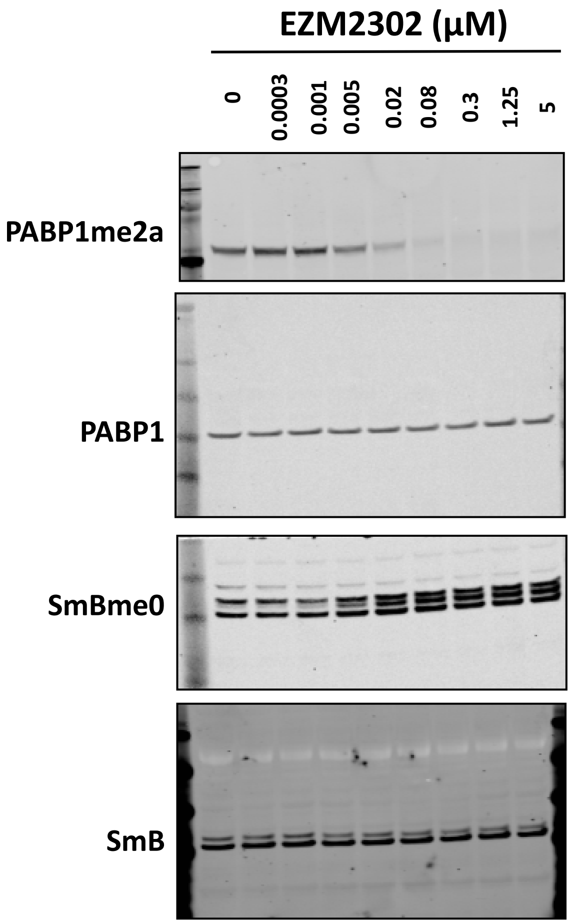

Supplementary Figure S6. Uncropped images from Supplementary Figures S3.

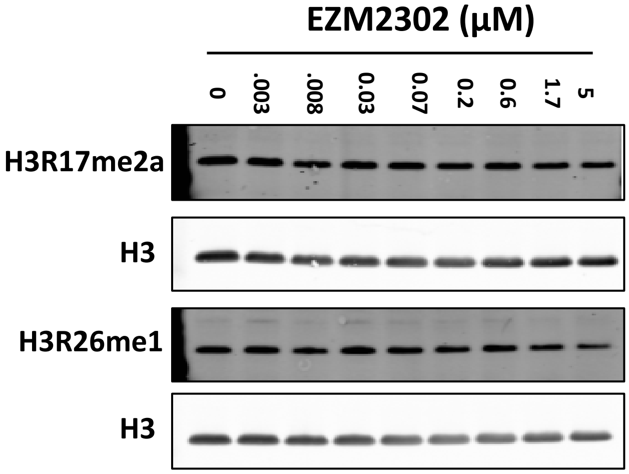

Supplementary Figure S7. Minimal effects of EZM2302 on histone methyl mark inhibition using western blot. H3R17me2a and H3R26me1 were minimally impacted after four days of EZM2302 treatment in RPMI-8226 cells. Cells were treated with a dose-titration of 0.0003 to 5 µM compound. IC_{50} values were greater than 20 µM.

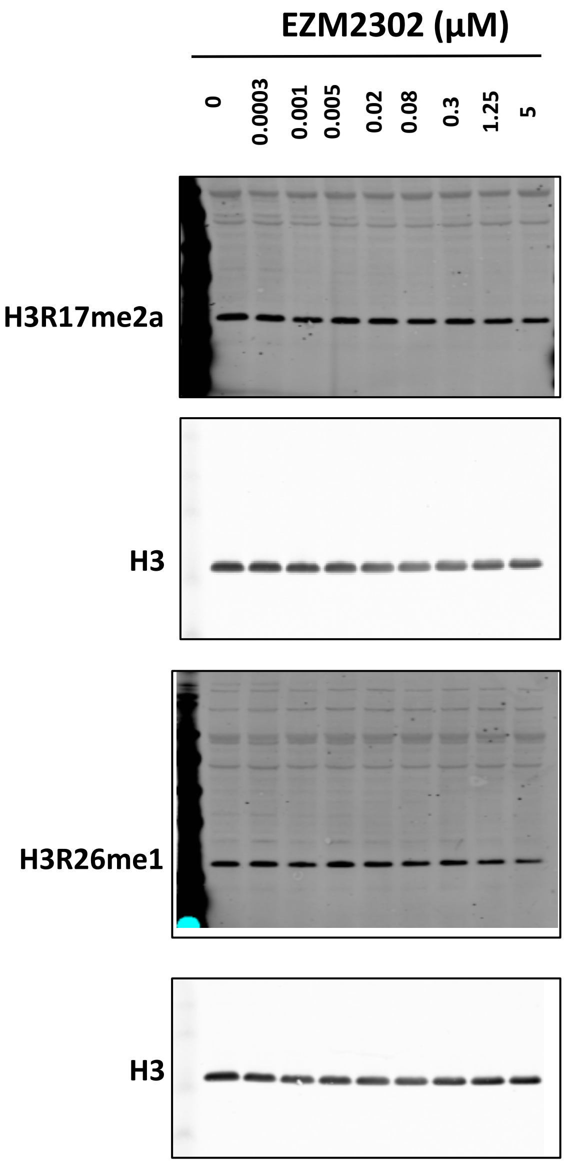

Supplementary Figure S8. Uncropped images from Supplementary Figure S7

a

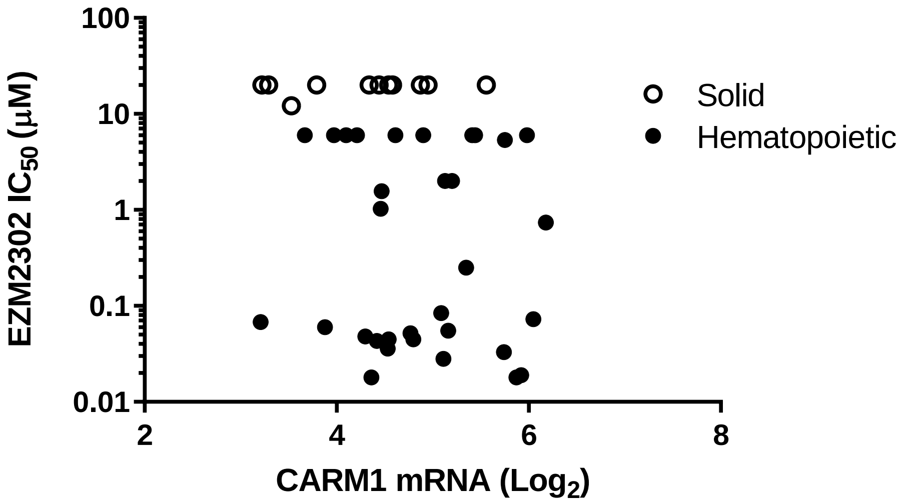

b

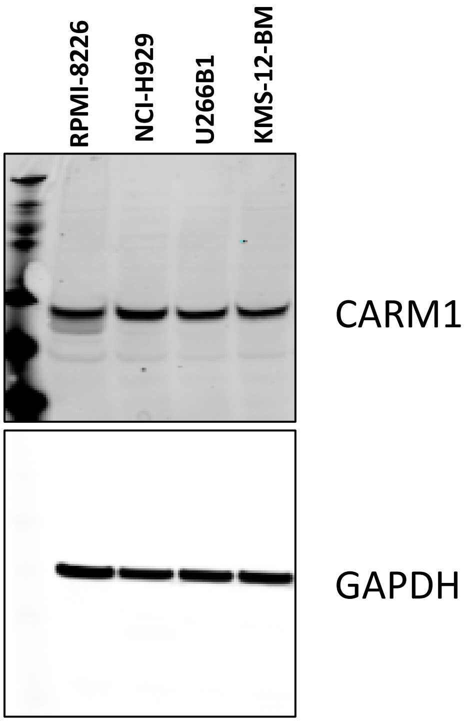

Supplementary Figure S9. CARM1 protein levels do not predict sensitivity to EZM2302. A. Anti-proliferative activity of EZM2302 (Day 15 Abs IC_{50}) is not related to CARM1 mRNA levels (Broad Institute's Cancer Cell Line Encyclopedia [10]). B. CARM1 protein levels are consistent across MM cell lines, including both lines sensitive and insensitive to EZM2302.

a

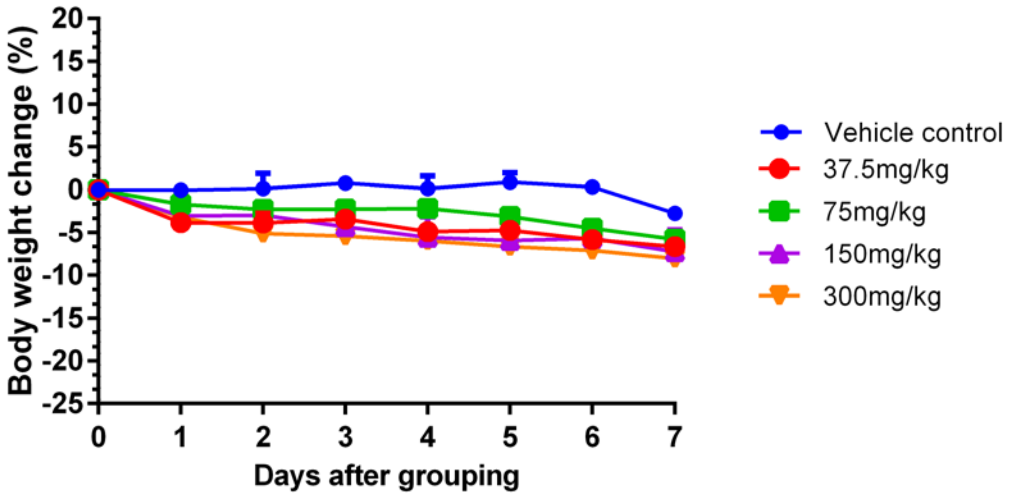

b

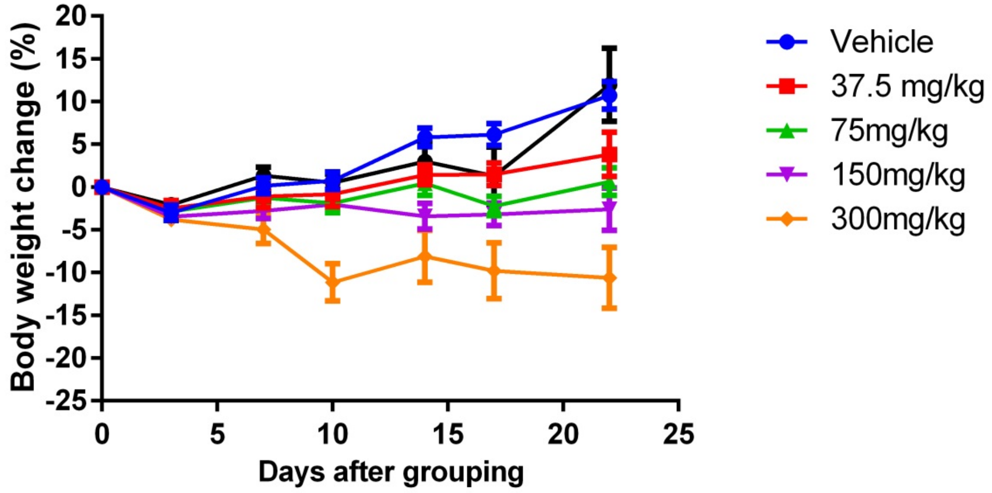

Supplementary Figure S10. EZM2302 is well tolerated in SCID mice. A, Bodyweight change (%) of SCID mice following EZM2302-treatment in 7 day dose-range finding study. Mean body weight changes induced by twice daily (BID) administration of EZM2302 for 7 days at the indicated doses (data shown as mean values ± SEM, n=3 mice per group). **B,** Bodyweight change (%) of mice following EZM2302-treatment in RPMI-8226 xenograft Mice in 21-day efficacy studies. Mean body weight changes induced by twice daily (BID) administration of EZM2302 for 21 days at the indicated doses. 6 of 8 animals in the 300 mg/kg group were put on dosing holidays for between 2 and 8 days due to weight loss of greater than 8%. Dosing resumed when body weight recovered. Compound administration for all animals was stopped on day 21, and tumors were harvested for PD analysis (data shown as mean values ± SEM, n=8 mice per group).

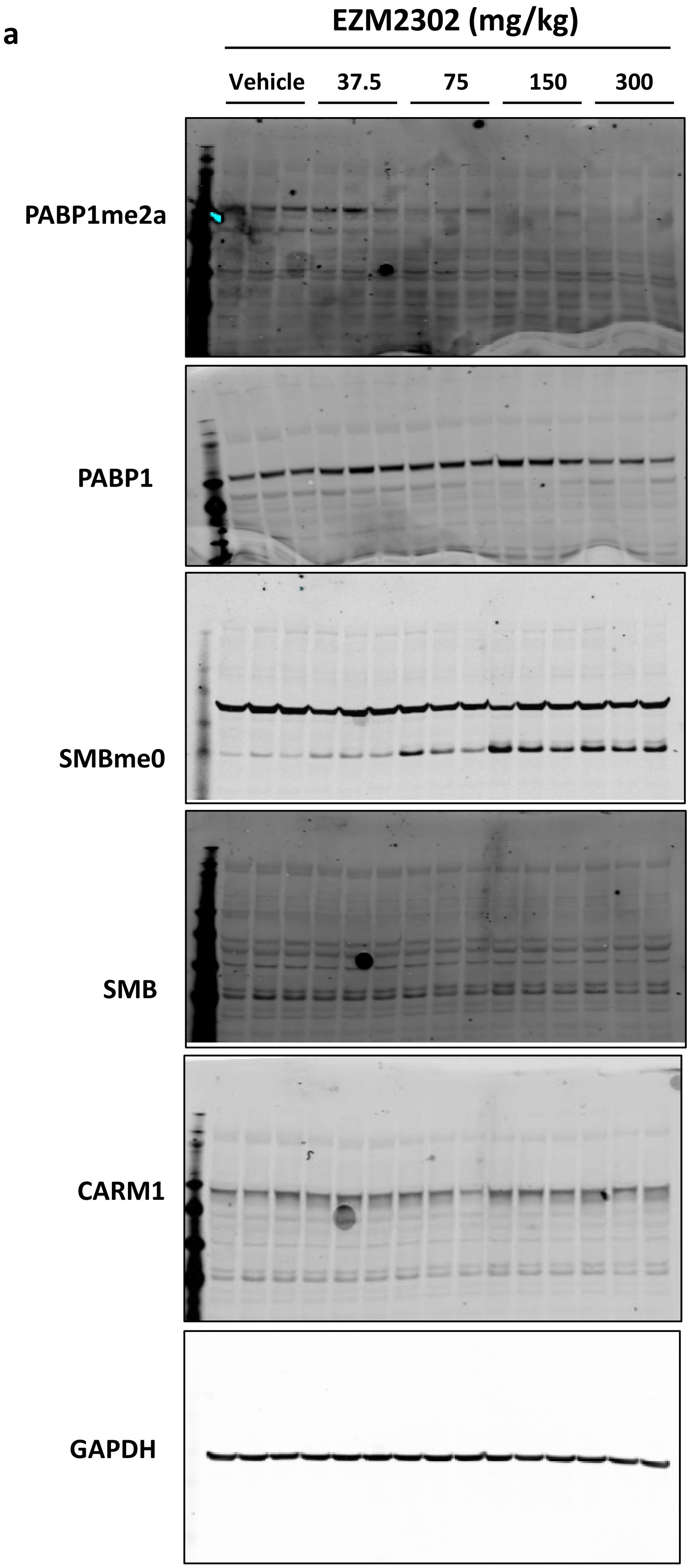

Supplementary Figure S11. Uncropped images from Figure 4 **A.** Uncropped images from Figure 4C. **B.** Uncropped images from Figure 4E.

b

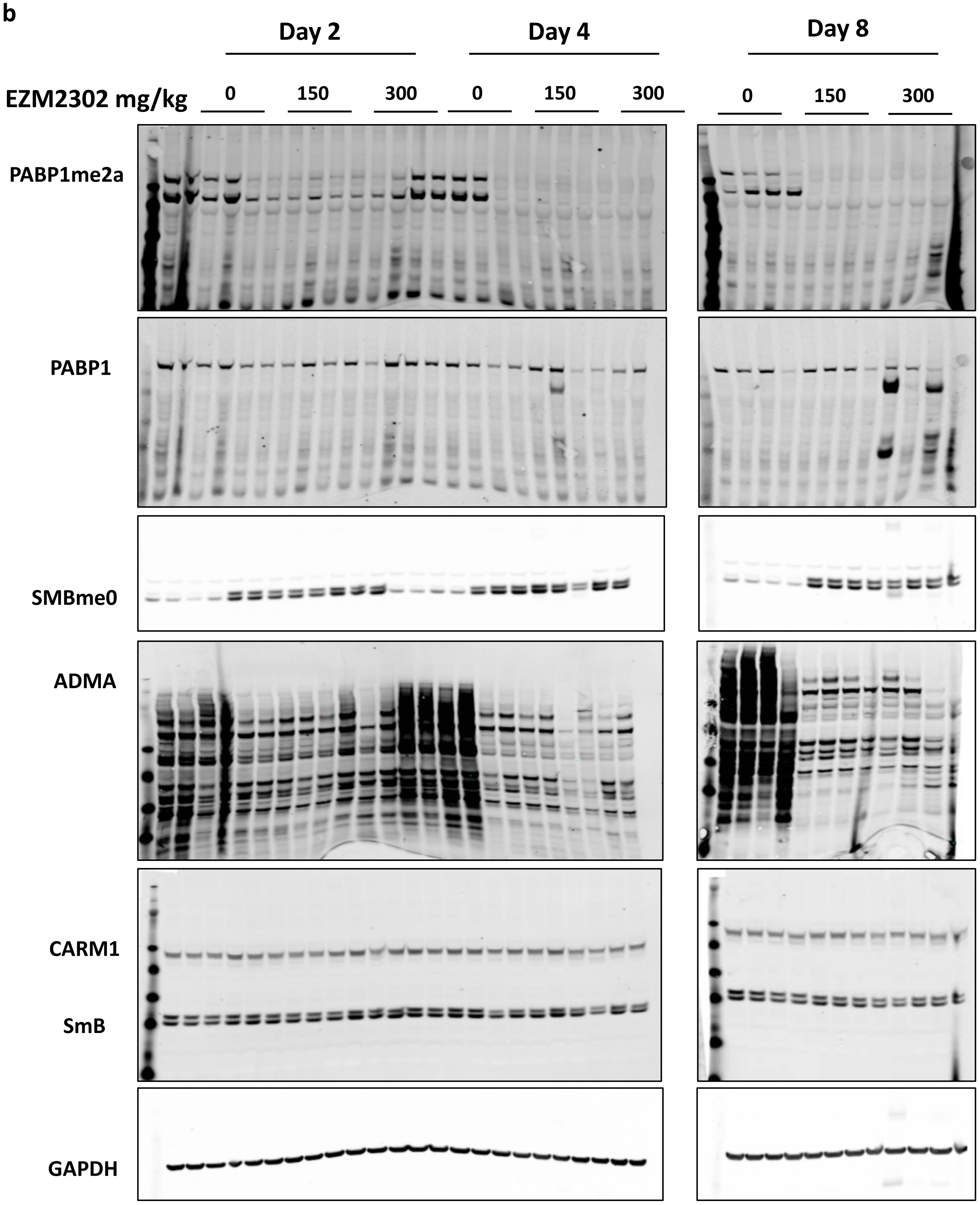

Supplementary Figure S12. Uncropped images from Figure 4. **A.** Uncropped images from Figure 4C. **B.** Uncropped images from Figure 4E.

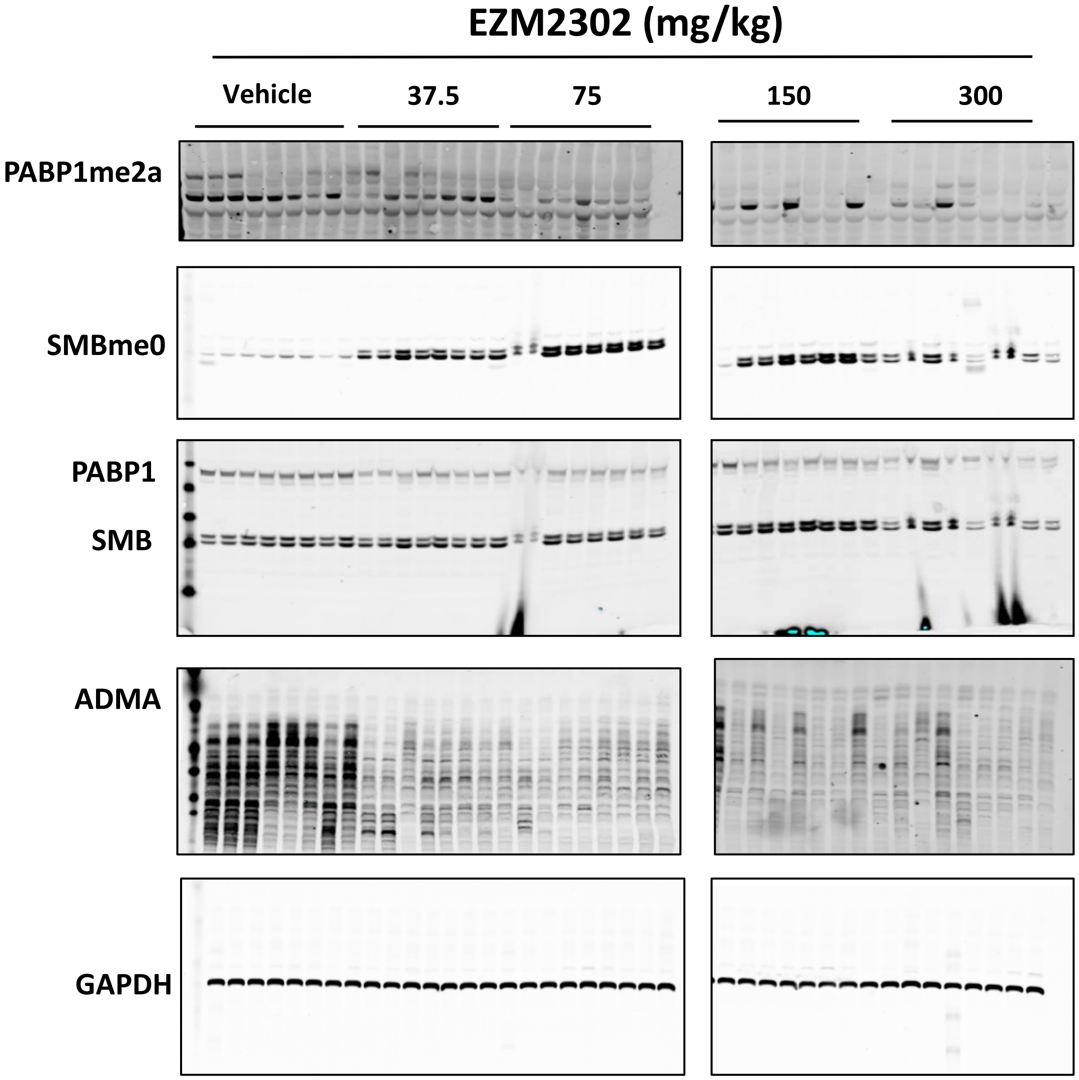

Supplementary Figure S13. Uncropped images from Figure 5.

REFERENCES

1 Kabsch, W. Xds. *Acta Crystallogr D Biol Crystallogr* **66**, 125-132, doi:10.1107/S0907444909047337 (2010).

2 Evans, P. Scaling and assessment of data quality. *Acta Crystallogr D Biol Crystallogr* **62**, 72-82, doi:10.1107/S0907444905036693 (2006).

3 Evans, P. R. & Murshudov, G. N. How good are my data and what is the resolution? *Acta Crystallogr D Biol Crystallogr* **69**, 1204-1214, doi:10.1107/S0907444913000061 (2013).

4 Schuttelkopf, A. W. & van Aalten, D. M. PRODRG: a tool for high-throughput crystallography of protein-ligand complexes. *Acta Crystallogr D Biol Crystallogr* **60**, 1355-1363, doi:10.1107/S0907444904011679 (2004).

5 Murshudov, G. N., Vagin, A. A. & Dodson, E. J. Refinement of macromolecular structures by the maximum-likelihood method. *Acta Crystallogr D Biol Crystallogr* **53**, 240-255, doi:10.1107/S0907444996012255 (1997).

6 Emsley, P., Lohkamp, B., Scott, W. G. & Cowtan, K. Features and development of Coot. *Acta Crystallogr D Biol Crystallogr* **66**, 486-501, doi:10.1107/S0907444910007493 (2010).

7 Copeland, R. A. *A Practical Introduction to Structure, Mechanism and Data Analysis*. (Wiley, 2000).

8 Riera, T. V., Wigle, T. J. & Copeland, R. A. Characterization of Inhibitor Binding Through Multiple Inhibitor Analysis: A Novel Local Fitting Method. *Methods Mol Biol* **1439**, 33-45, doi:10.1007/978-1-4939-3673-1_2 (2016).

9 Knutson, S. K. *et al.* A selective inhibitor of EZH2 blocks H3K27 methylation and kills mutant lymphoma cells. *Nat Chem Biol* **8**, 890-896, doi:10.1038/nchembio.1084 [pii] (2012).

10 Barretina, J. *et al.* The Cancer Cell Line Encyclopedia enables predictive modelling of anticancer drug sensitivity. *Nature* **483**, 603-607, doi:10.1038/nature11003 (2012).
